# A self-amplifying RNA vaccine against COVID-19 with long-term room-temperature stability

**DOI:** 10.1038/s41541-022-00549-y

**Published:** 2022-11-02

**Authors:** Emily A. Voigt, Alana Gerhardt, Derek Hanson, Madeleine F. Jennewein, Peter Battisti, Sierra Reed, Jasneet Singh, Raodoh Mohamath, Julie Bakken, Samuel Beaver, Christopher Press, Patrick Soon-Shiong, Christopher J. Paddon, Christopher B. Fox, Corey Casper

**Affiliations:** 1 RNA Vaccines, Access to Advanced Health Institute (AAHI), Seattle, WA 98102 USA; 2 Product Development, Access to Advanced Health Institute (AAHI), Seattle, WA 98102 USA; 3 Formulation Sciences, Access to Advanced Health Institute (AAHI), Seattle, WA 98102 USA; 4 ImmunityBio, Inc, Culver City, CA 90232 USA; 5 Amyris, Inc, Emeryville, CA 94608 USA; 6 Department of Global Health, University of Washington, Seattle, WA 98195 USA; 7 Department of Medicine, University of Washington, Seattle, WA 98195 USA; 8 Vaccine and Infectious Disease Division, Fred Hutchinson Cancer Center, Seattle, WA 98109 USA

**Keywords:** RNA vaccines, RNA vaccines

## Abstract

mRNA vaccines were the first to be authorized for use against SARS-CoV-2 and have since demonstrated high efficacy against serious illness and death. However, limitations in these vaccines have been recognized due to their requirement for cold storage, short durability of protection, and lack of access in low-resource regions. We have developed an easily-manufactured, potent self-amplifying RNA (saRNA) vaccine against SARS-CoV-2 that is stable at room temperature. This saRNA vaccine is formulated with a nanostructured lipid carrier (NLC), providing stability, ease of manufacturing, and protection against degradation. In preclinical studies, this saRNA/NLC vaccine induced strong humoral immunity, as demonstrated by high pseudovirus neutralization titers to the Alpha, Beta, and Delta variants of concern and induction of bone marrow-resident antibody-secreting cells. Robust Th1-biased T-cell responses were also observed after prime or homologous prime-boost in mice. Notably, the saRNA/NLC platform demonstrated thermostability when stored lyophilized at room temperature for at least 6 months and at refrigerated temperatures for at least 10 months. Taken together, this saRNA delivered by NLC represents a potential improvement in RNA technology that could allow wider access to RNA vaccines for the current COVID-19 and future pandemics.

## Introduction

More than 609 million confirmed cases of coronavirus disease 2019 (COVID-19) and 6.5 million related deaths have occurred worldwide due to severe acute respiratory syndrome coronavirus 2 (SARS-CoV-2) as of September 2022^1^. Although multiple SARS-CoV-2 vaccines have been developed and over 12 billion SARS-CoV-2 vaccine doses have been administered globally^1^, only 21% of people in low-income countries have received a dose^2^. New vaccines that can be manufactured rapidly and stored without complex cold-chain requirements and result in long-term, robust, multi-variant immunity are urgently needed to protect people across the globe against SARS-CoV-2.

Since the emergence of SARS-COV-2 in 2019, two mRNA vaccines, Moderna mRNA-1273 and Pfizer-BioNTech BNT162b2, were developed most rapidly and proved to be most efficacious^3–5^. These vaccines use nucleoside-modified mRNAs encoding the prefusion-stabilized spike protein of SARS-CoV-2, which are encapsulated by lipid nanoparticles (LNPs) for stabilization and intracellular delivery^6,7^. However, global access to these COVID-19 vaccines has been severely hampered by the complex cold-chain requirements for mRNA vaccines and limited vaccine distribution in low- and middle-income countries^8^. Unopened vials of the currently administered mRNA/LNP vaccines, mRNA-1273 and BNT162b2, are currently licensed for use within very restrictive temperature conditions: for 9 months frozen (−25 to −15 °C or −90 to −60 °C, respectively), 1 month refrigerated (2–8 °C), and 24 or 4 h at room temperature, respectively^9,10^. While efforts to improve the thermostability of LNP-based vaccines are progressing^11–15^, non-frozen distribution and storage have yet to be realized with a licensed mRNA vaccine product. Despite efforts, long-term stability of mRNA/LNP systems at non-frozen temperatures may be difficult to achieve due to the susceptibility of mRNA to cleavage and the inherent complexity of the LNP delivery system^16,17^, and stability is limited even under frozen or dried conditions^17–19^.

Improvements to RNA vaccines are needed to increase stability during storage at temperatures above freezing and to streamline manufacturing processes for global distribution^20,21^. Additionally, current mRNA/LNP vaccine technology requires proprietary ionizable lipids, a consistent supply of GMP raw materials for LNP production, and complex manufacturing processes that are difficult to scale worldwide for a pandemic response^22^. Waning of the immune responses to these authorized mRNA/LNP vaccines and the continued emergence of SARS-CoV-2 variants of concern are now necessitating the use of regular booster doses^23–25^, further hampering full protection of individuals in low-resource countries^26^. Development of a more potent, easily manufactured, and thermostable RNA vaccine against COVID-19 is necessary to enable global access and a comprehensive pandemic response.

Self-amplifying RNA (saRNA) vaccines—in which RNAs encode viral replicase genes in addition to antigen genes—promise improved potency and durability due to both the amplification of the RNA in vivo after delivery and the adjuvanting properties of dsRNA and replication intermediates^16,27,28^. Indeed, saRNAs have been successfully used to create COVID-19 vaccines^29–35^ that have reached clinical trials^36,37^. However, thermostability of these vaccine formulations is either unknown^29,30,32–35^ or 1 week at refrigerated or room-temperature conditions^31^. Thus, further evaluation of the stability of saRNA vaccines is warranted, although it is unlikely that their storage stability is markedly better than mRNA vaccines using the same delivery platform.

We previously developed a lyophilizable, thermostable saRNA vaccine platform with high potency^38^ and long-term storage capability^39^. This platform is based on a unique, highly manufacturable nanostructured lipid carrier (NLC)^38^ that allows for lyophilization of the drug product, which can extend shelf life to 21 months in refrigerated conditions and 8 months at room temperature^39^. In the current study, we have applied this thermostable saRNA vaccine platform to COVID-19, efficiently expressing SARS-CoV-2 spike protein antigens and driving induction of robust humoral and cellular SARS-CoV-2-specific immune responses in mice without the need for modified nucleosides. This formulation is thermostable when lyophilized, maintaining immunogenicity for at least 6 months at room temperature or standard refrigerated conditions. Taken together, this potent and shelf-stable RNA vaccine platform could be an important contribution to increasing global access to COVID-19 vaccines, especially in low-resource regions.

## Results

### saRNA/NLC SARS-CoV-2 vaccine characterization and antigen expression

**Fig. 1:**
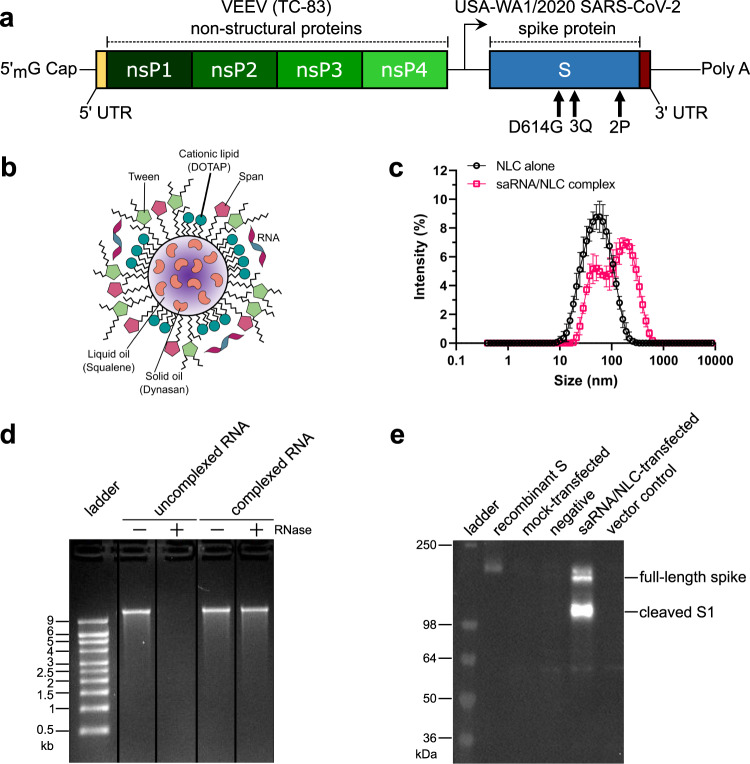
Design and characterization of SARS-CoV-2 D614G saRNA/NLC vaccine. **a** SARS-CoV-2 saRNA vaccine schematic. **b** RNA/nanostructured lipid carrier (NLC) vaccine formulation. Design by Cassandra Baden. **c** Size distribution of NLC formulation particles alone (black) and saRNA/NLC complex (pink) showing mean and standard deviation of *n* = 3 replicate measurements for each sample. **d** SARS-CoV-2 saRNA complexed to the outside of the NLC particles is full-length intact saRNA that is protected by the NLC complexation from RNase degradation. Lanes were derived from the same gel and re-arranged (see Supplementary Fig. 7). **e** Western blot showing SARS-CoV-2 spike (S) protein expression in saRNA/NLC vaccine-transfected HEK-293 cells. Blot was derived from the same experiment and processed in parallel. See Supplementary Fig. 7 for the original unprocessed blot image.

Our baseline saRNA/NLC SARS-CoV-2 vaccine candidate (designated D614G) consisted of the codon-optimized Wuhan-D614G spike protein sequence^40^ (Fig. 1a). This vaccine saRNA was complexed with an NLC delivery nanoparticle by simple mixing to create the vaccine complex with the RNA bound to the exterior of the NLC nanoparticle (Fig. 1b) and with an average hydrodynamic particle size of 89 ± 3 nm (Fig. 1c). Complexing the saRNA with NLC allowed for retention of RNA integrity even in the presence of RNase (Fig. 1d, Supplementary Fig. 7a). The formulated vaccine was then transfected into HEK-293 cells where it demonstrated significant SARS-CoV-2 spike protein expression 24 hours post-transfection as measured by western blot (Fig. 1e, Supplementary Fig. 7b). In order to mitigate supply chain issues for the least readily available ingredient of the NLCs, the cationic lipid DOTAP, we also tested DOTAP from multiple independent manufacturers and demonstrated similar physicochemical properties and 3-month stability of the resulting NLCs, indicating that material to supply large-scale manufacturing may likely be successfully sourced from multiple vendors (Supplementary Table 1).

### Baseline saRNA/NLC vaccine with the D614G construct induces strong Th1-biased neutralizing antibody responses in mice

**Fig. 2:**
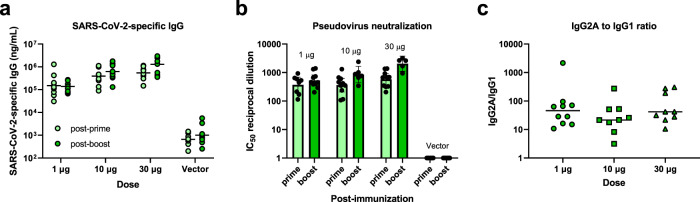
Immunogenicity of SARS-CoV-2 D614G saRNA/NLC vaccine after prime or prime-boost immunization of C57BL/6J mice. **a** Serum SARS-CoV-2 spike protein-binding IgG. Horizontal lines show geometric mean. **b** Serum SARS-CoV-2 neutralizing antibodies against the original Wuhan strain. Results show geometric mean and 95% confidence interval. **c** Serum SARS-CoV-2 spike protein-binding IgG1 versus IgG2a indicates a strong Th1-biased response. Horizontal lines show median. The vector control represents mice injected with 10 μg of NLC-complexed saRNA expressing the non-immunogenic secreted embryonic alkaline phosphatase gene. *n* = 10 mice per group.

To test vaccine immunogenicity of the baseline D614G vaccine candidate, complexed saRNA/NLC was injected intramuscularly into C57BL/6J mice at 1, 10, or 30 µg doses, with a boost dose 3 weeks post-prime. As a negative vector control, an saRNA expressing the non-immunogenic reporter secreted embryonic alkaline phosphatase (SEAP) gene in the place of the SARS-CoV-2 spike gene was used at a 10 µg dose. Serum samples were taken 21 days post-prime and 21 days post-boost. These samples were assessed for SARS-CoV-2 spike protein-binding IgG, IgG1, and IgG2a antibodies by enzyme-linked immunosorbent assay (ELISA) and for SARS-CoV-2 neutralizing antibodies by pseudovirus neutralization assay (Fig. 2).

Strong antibody responses to SARS-CoV-2 spike protein were detected in all vaccinated mice post-prime (Fig. 2a), with a statistically significant but relatively small rise in titers after the boost dose (*p* = 0.0199, mixed-effects analysis). A clear dose dependency was seen across the mouse groups. Strong neutralization of Wuhan-D614G pseudotyped lentiviral particles was evident in all vaccinated mouse sera post-prime and post-boost (Fig. 2b), with no detectable neutralization noted in vector control-vaccinated mouse sera. Ratios of IgG2a (Th1) to IgG1 (Th2) antibody titers indicated a robust Th1 bias in the humoral responses to the baseline D614G saRNA/NLC vaccine (Fig. 2c).

### Antigen optimization increases SARS-CoV-2 variant cross-neutralization

**Fig. 3:**
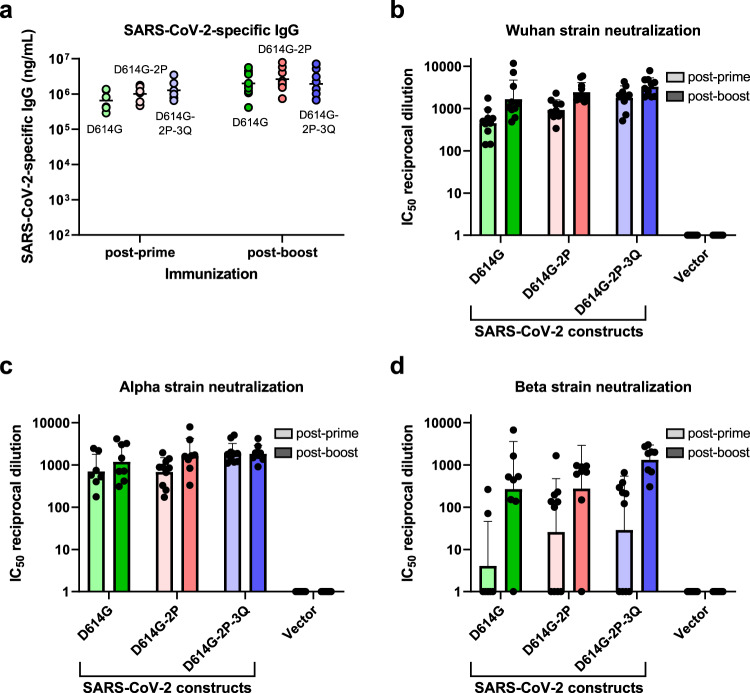
Immunogenicity of SARS-CoV-2 D614G, D614G-2P, and D614G-2P-3Q saRNA/NLC vaccines after 10 µg prime or prime-boost immunization of C57BL/6J mice. **a** Serum SARS-CoV-2 spike protein-binding IgG. Horizontal lines show geometric mean. **b–d** Serum SARS-CoV-2 neutralizing antibody titers against the original Wuhan, Alpha (B.1.1.7), and Beta (B.1.351) pseudovirus variants. The vector control represents mice injected with 10 μg of NLC-complexed saRNA expressing the non-immunogenic secreted embryonic alkaline phosphatase gene. Results show geometric mean and geometric standard deviation. *n* = 10 mice per group.

We next sought to optimize the SARS-CoV-2 spike protein sequence for the induction of SARS-CoV-2 antibodies capable of neutralizing multiple variants of the virus. C57BL/6J mice were injected intramuscularly with 10 µg of saRNA/NLC vaccine expressing the native D614G spike protein sequence, the D614G spike protein sequence with a diproline mutation that stabilized the prefusion conformation of the spike trimer^41,42^ (designated D614G-2P), or the D614G diproline-stabilized spike protein with a QQAQ mutation that ablated the furin cleavage site^43^ (designated D614G-2P-3Q). Each saRNA candidate was complexed with NLC in the same manner as with the baseline candidate, and similar vaccine particle size, RNA integrity, and in vitro spike protein expression were observed as in Fig. 1 (Supplementary Figs. 1, 9). Mice were again administered prime and boost injections 3 weeks apart, and serum samples were taken 3 weeks post-prime and 3 weeks post-boost vaccine dose. All vaccine candidates were well tolerated based on mouse general appearance and behavior, with no signs of weight loss or injection site reactogenicity, similar to previous studies with this saRNA/NLC platform^38,39,44,45^. SARS-CoV-2 spike-binding IgG antibodies were assessed in mouse sera by ELISA, and SARS-CoV-2 variant neutralization was evaluated by pseudovirus neutralization assay (Fig. 3).

All three vaccine candidates induced SARS-CoV-2 spike-binding serum IgG titers at high levels post-prime, with a significant increase post-boost (Fig. 3a, average 2.7-fold change post-boost, *p* = 0.0021 across groups, mixed-effects analysis). No significant difference in spike-binding IgG titers was seen between the three candidates post-boost (*p* = 0.6606, one-way ANOVA). The candidates differed only in their ability to consistently induce antibodies in all mice that cross-neutralize emerging SARS-CoV-2 variants (Fig. 3b-d). All three candidates largely retained the neutralization capacity to the Alpha variant (B.1.1.7, UK). However, cross-neutralization against the Beta (B.1.351, South African) variant decreased significantly in D614G- and D614G-2P-vaccinated mouse sera (2.3-fold [*p* = 0.0063] and 4.5-fold [*p* = 0.0011] change post-prime, respectively, mixed-effects analysis with multiple comparisons). This reduction in neutralizing capacity was smaller in sera from D614G-2P-3Q-vaccinated mice and no longer statistically significant (2.2-fold change, *p* = 0.1606, mixed-effects analysis with multiple comparisons), indicating improved induction of variant cross-neutralizing antibodies by this vaccine candidate. When produced with modified nucleosides rather than standard nucleosides, in a brief study to determine whether use of modified uracil affects saRNA vaccine immunogenicity as it does standard mRNA vaccines, this vaccine’s ability to induce antibody and T cell responses in mice was entirely abrogated (Supplementary Fig. 2) rather than enhanced. The standard-nucleoside D614G-2P-3Q saRNA/NLC vaccine candidate was therefore chosen as the lead vaccine candidate for further immunogenicity studies and is hereafter referred to as “AAHI-SC2.”

### Optimized saRNA/NLC vaccine AAHI-SC2 induces strong variant cross-protective immune responses and establishment of bone marrow-resident antibody-secreting cell populations

**Fig. 4:**
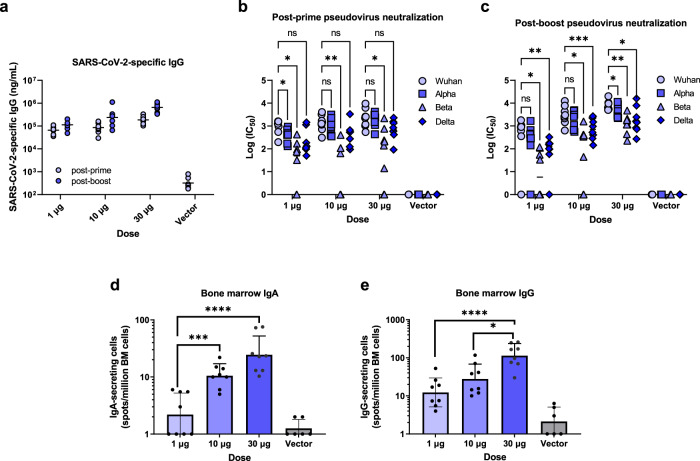
Humoral immunogenicity profiles of optimized SARS-CoV-2 D614G-2P-3Q saRNA/NLC vaccine (AAHI-SC2) after prime or prime-boost immunization of C57BL/6 J mice. **a** Serum SARS-CoV-2 spike protein-binding IgG. Horizontal lines show geometric mean. Serum SARS-CoV-2 neutralizing antibody titers post-prime (**b**) and post-boost (**c**). Horizontal lines show geometric mean. Data were log-transformed and evaluated by mixed effects analysis with multiple comparisons. Induction of bone marrow (BM)-resident IgA- (**d**) and IgG-secreting (**e**) cells by ELISpot. Results show geometric mean and geometric standard deviation. Analyzed with one-way ANOVA with multiple comparisons. *p < 0.05, **p < 0.01, ***p < 0.001, ****p < 0.0001. The vector control re*p*resents mice injected with 10 μg of NLC-complexed saRNA expressing the non-immunogenic secreted embryonic alkaline phosphatase (SEAP) gene. *n* = 6 mice for SEAP control group and *n* = 8 for dosing groups.

After selecting AAHI-SC2 as lead vaccine candidate, we conducted a vaccine dosing study in mice with comprehensive humoral and cellular immunogenicity characterization. C57BL/6J mice were immunized in prime or prime-boost regimens with AAHI-SC2 at doses of 1, 10, or 30 µg. Serum samples were collected to measure SARS-CoV-2 spike-specific IgG (Fig. 4a) and neutralizing antibody titers (Fig. 4b, c). Bone marrow samples were taken post-boost and used for measurement of SARS-CoV-2 spike-specific IgA- and IgG-secreting cells by ELISpot (Fig. 4d, e).

High levels of SARS-CoV-2 IgG antibodies were induced in mouse sera in a dose-dependent manner, with even a 1 µg dose inducing high responses (Fig. 4a). Boost vaccine doses increased IgG titers a small but significant degree at all doses (average threefold change, *p* < 0.0001, two-way ANOVA). Sera were able to neutralize Wuhan, Alpha (B.1.1.7), Beta (B.1.351), and Delta (B.1.617.2) pseudoviruses at high levels, both after prime-only and prime-boost vaccination (Fig. 4b, c). A significant decrease in the ability of the mouse sera to neutralize the Beta and Delta variants was evident relative to Wuhan strain neutralization as expected both post-prime (average *p* < 0.0001 and *p* = 0.0002, respectively, two-way ANOVA) and post-boost (*p* = 0.0006 and *p* < 0.0001, respectively, two-way ANOVA); however, neutralization of all three variants still generally remained at high, detectable levels. High levels of post-boost bone marrow-resident IgA-secreting and IgG-secreting cells were detected (Fig. 4d, e), suggesting induction of at least low levels of mucosal immune responses by the vaccine as well as establishment of robust long-lived IgG-secreting plasma cell populations.

### Optimized AAHI-SC2 vaccine induces robust Th1-biased cellular immune responses

**Fig. 5:**
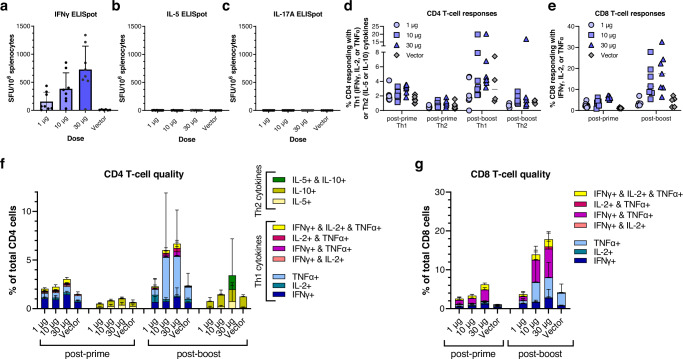
Detailed cellular immunogenicity profiles of optimized SARS-CoV-2 D614G-2P-3Q saRNA/NLC vaccine (AAHI-SC2) after prime or prime-boost immunization of C57BL/6 J mice. **a** SARS-CoV-2 spike-reactive spleen-resident IFNγ^+^ T cells by ELISpot. SFU = spot forming units. Results show mean and standard deviation (SD). **b** SARS-CoV-2 spike-reactive spleen-resident IL-5^+^ T cells by ELISpot. Results shows mean and SD. **c** SARS-CoV-2 spike-reactive spleen-resident IL-17-A^+^ T cells by ELISpot. Results shows mean and SD. CD4 (**d**) and CD8 (**e**) T cells responding with any Th1 (IFNγ, IL-2, or TNFα) or Th2 (IL-5 or IL-10) cytokines post-prime and post-boost were plotted representing the total number of responding cells, for CD4 or CD8 cells per mouse. Horizontal lines show median. **f**, **g** Quality of responding CD4 and CD8 T cells. Total Th1 or Th2 responses were subdivided by cells responding with one, two, or three cytokine(s) to show magnitude and quality of Th1 and Th2 responses, with bar graphs showing the average percentage across each group of mice. The vector control represents mice injected with 10 μg of NLC-complexed saRNA expressing the non-immunogenic secreted embryonic alkaline phosphatase (SEAP) gene. Results show mean and SD. *n* = 6 mice for SEAP control group and *n* = 8 for dosing groups.

We also characterized cellular responses after prime-only or prime-boost vaccination by stimulation of splenocytes with SARS-CoV-2 overlapping peptides followed by T cell ELISpot (Fig. 5a-c) or intracellular cytokine staining and flow cytometry (Fig. 5d-g, Supplementary Fig. 3). Robust Th1-biased T cell responses were seen in vaccinated mice. T cell ELISpots demonstrated large numbers of IFNγ-secreting cells after stimulation with SARS-CoV-2 spike protein overlapping peptides even after a prime dose alone, with clear vaccine dose-dependent responses evident (Fig. 5a). IL-5- (Fig. 5b) and IL-17A-secreting (Fig. 5c), Th2-type T cells were induced at very low or undetectable levels, indicating a strong Th1 bias in cellular responses to AAHI-SC2 vaccination.

Flow cytometry of splenocytes stimulated with SARS-CoV-2 spike overlapping peptides and stained for a panel of T cell markers and cytokines generated results consistent with the T cell ELISpot findings. High levels of responsive CD4^+^ Th1 and robust CD8^+^ T cell populations were stimulated with prime vaccination alone, and these were increased several-fold in a dose-dependent manner after boost injection. Polyfunctional, triple-positive IFNγ-, IL-2-, and TNFα-secreting CD4^+^ (Fig. 5d) and CD8^+^ T cells (Fig. 5e) were stimulated at whole-percentage levels after a prime dose alone and remained high post-boost. Large populations of IFNγ^+^ and TNFα^+^ cells post-prime, increasing to a large degree post-boost, drove a significant SARS-CoV-2-specific significant CD4 Th1 response and a large CD8 T cell response (Fig. 5f, g), representing up to 20% of the CD8 cell population in the highest-dosed group. Conversely, the CD4 Th2 response was minimal at all timepoints, generally remaining under 2% of CD4 cells, and only measurable at the highest vaccine doses (Fig. 5f).

### Lyophilized AAHI-SC2 vaccine is thermostable, enabling long-term storage

Short-term stability of the liquid AAHI-SC2 vaccine in an excipient background of 20% w/v sucrose and 5 mM sodium citrate was evaluated over 2 weeks of storage at −80 °C, −20 °C, 2–8 °C, 25 °C, and 40 °C and compared to freshly prepared controls. Vaccine formulation particle size (70–100 nm) was maintained over the 2-week period at all storage temperatures (Supplementary Fig. 4a). The vaccine also retained its ability to induce SARS-CoV-2-specific serum IgG in C57BL/6 mice as measured by ELISA after storage at all temperatures except 40 °C (Supplementary Fig. 4b) and showed a trend towards potentially reduced potency developing in the 25 °C-stored vaccines after 2 weeks.

**Fig. 6:**
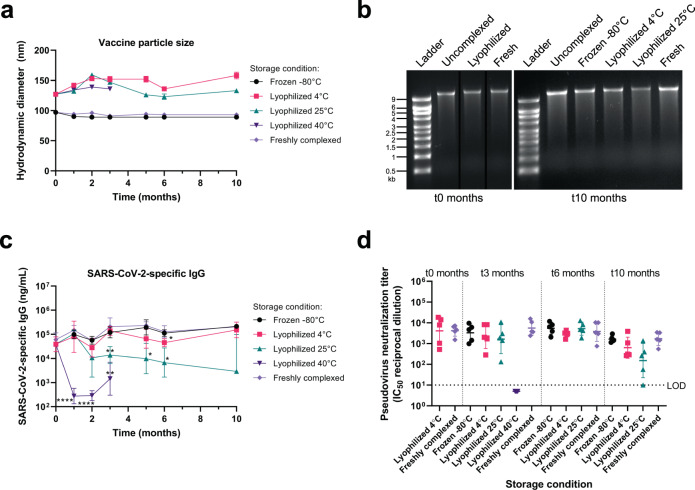
Stability of the lyophilized SARS-CoV-2 saRNA/NLC vaccine (AAHI-SC2) after 10 months of storage at different temperatures. **a** Mean hydrodynamic (Z-average) diameter of the vaccine complex with error bars indicating the standard deviation (SD) of *n* = 3 replicate measurements for each condition at each timepoint. **b** Integrity of vaccine RNA at 0 and 10 months of storage at the indicated temperatures. “Uncomplexed” refers to saRNA alone that had been stored at −80 °C for the indicated length of time. Lanes were derived from the same gel and re-arranged for the 0 months of storage image. See Supplementary Fig. 8 for original unprocessed gel images. **c** Serum SARS-CoV-2 spike protein-binding IgG induced in female C57BL/6J mice by the vaccine after storage under the indicated conditions for the indicated times. *n* = 5 mice per condition per timepoint. Antibody plots show geometric mean and geometric SD. Statistical analysis conducted on log-transformed data by Welch’s ANOVA test with Dunnett’s T3 multiple comparison test at each timepoint, comparing each stored vaccine preparation to freshly complexed vaccine. *p < 0.05, **p < 0.01, ****p < 0.0001. **d** Pseudovirus neutralization titers induced in female C57BL/6 J mice by the vaccine after storage under the indicated conditions for the indicated times. LOD = limit of detection. *n* = 5 mice per condition per timepoint. Results show geometric mean and geometric SD. Statistical analysis conducted on log-transformed data by Welch’s ANOVA test with Dunnett’s T3 multiple comparison test at each timepoint, comparing each stored vaccine preparation to freshly complexed vaccine.

In order to achieve long-term storage stability, we lyophilized the AAHI-SC2 vaccine and characterized it before and after storage at various temperatures compared to the frozen liquid vaccine. Previously, we demonstrated proof of concept for the long-term storage stability of this platform using SEAP reporter saRNA in the presence of 20% w/v sucrose as a lyoprotectant with a background of 2 mM sodium citrate (and trace amounts of Tris buffer from the bulk RNA material)^39^. Here, the AAHI-SC2 vaccine was lyophilized under those same conditions in addition to two alternative excipient backgrounds: 20% sucrose with 5 mM sodium citrate (increasing the concentration of sodium citrate, a metal chelator) and 20% sucrose with 1 mM sodium citrate and 10 mM Tris (increasing the pH of the system with addition of Tris buffer). These alternative excipient backgrounds were selected based on conditions that are commonly used for the storage of RNA alone either in the literature or in commercially available RNA products and also contained approximately 2.6 mM Tris buffer from the bulk RNA material. Data with the alternative excipient backgrounds are presented in Supplementary Figs. 5, 10; however, the original proof-of-concept excipient formulation performed the best (Fig. 6).

Vaccine at each excipient condition was stored frozen at −80 °C or lyophilized at 4 °C, 25 °C, or 40 °C. A freshly complexed positive control using saRNA that had been stored at −80 °C in 10 mM Tris pH 8 and NLC stored at 4 °C in 10 mM sodium citrate—conditions known to provide excellent stability for each^39^—was also prepared at each timepoint for comparison. For the proof-of-concept excipient formulation, vaccine particle size increased by ~30 nm immediately after lyophilization, but after that initial increase, particle size was maintained over the 10 months of the study (Fig. 6a). Measured at the end of the study, no meaningful difference in particle surface charge (zeta potential) of the proof-of-concept formulation was observed between stored samples and the freshly complexed control (Supplementary Fig. 6). RNA integrity was also maintained immediately after lyophilization and throughout 10 months of storage under frozen conditions and lyophilized conditions stored at 4 °C, but the lyophilized sample stored at 25 °C exhibited decreased integrity by gel electrophoresis (Fig. 6b, Supplementary Fig. 8). The accelerated storage condition of 40 °C was eliminated from the stability study after 3 months due to complete degradation of the RNA (Supplementary Fig. 10). As a control, saRNA alone that had been stored frozen at −80 °C was also evaluated and shown to maintain its integrity after storage.

Vaccine immunogenicity during storage was assessed by injecting stored vaccine intramuscularly into 6–8 week-old C57BL/6J mice (5 mice per storage condition per timepoint), collecting mouse serum samples 14 days post-immunization, and testing these sera for SARS-CoV-2 spike protein-binding antibodies by ELISA, as described above. Statistical analysis was conducted on log-transformed data by Welch’s ANOVA test with Dunnett’s T3 multiple comparison test at each timepoint, comparing each stored vaccine preparation to freshly complexed vaccine. The ability of each stored vaccine to induce an immune response in vivo was predicted well by the presence of a major intact RNA band by agarose gel electrophoresis. As expected, the −80 °C-frozen vaccine maintained the ability to induce SARS-CoV-2-specific IgG throughout the 10-month stability study equivalent to those induced by the freshly complexed vaccine (Fig. 6c) along with equivalent pseudovirus neutralization titers to freshly complexed vaccine (*p* > 0.05) (Fig. 6d). After storage at 40 °C for 3 months, both antibody titers and neutralizing titers were diminished with lyophilized vaccine (Fig. 6c, d), as expected for this accelerated degradation condition.

The lyophilized vaccine stored at 4 °C or at 25 °C continued to elicit serum SARS-CoV-2-specific-IgG titers throughout the study (Fig. 6c). The lyophilized 4 °C-stored vaccine maintained its ability to stimulate equivalent mouse serum IgG over the course of the 10-month study that was comparable to the lyophilized vaccine prior to storage and comparable to the freshly complexed control. The 25 °C-stored vaccine after 3 months of storage induced a decreased but still robust mouse serum IgG titer relative to both the lyophilized vaccine prior to storage and the freshly complexed vaccine at each timepoint (*p* < 0.05), and this decrease was consistent after 6 and 10 months of storage. Despite this apparent decrease in total spike-binding IgG after 6 months of storage at 25 °C, the lyophilized vaccine induced equivalent serum pseudovirus neutralization titers at 6 months, and only began to decline relative to fresh-mixed vaccine at 10 months of storage. Neutralizing antibody titers were maintained by the lyophilized vaccine stored at 4 °C throughout the 10-month study, showing no significant differences from the freshly-complexed or frozen vaccine (*p* > 0.05) (Fig. 6d). This suggests the long-term retention of lyophilized vaccine immunogenicity after storage at refrigerated temperatures and a lengthy (months-long) durability of efficacy if stored even at ambient temperatures.

## Discussion

AAHI-SC2, combining the potency and self-adjuvanting nature of saRNA with the excellent stability and immune-stimulating properties of the NLC formulation, exhibits many characteristics of a “next-generation” SARS-CoV-2 RNA vaccine. Immunization of mice with AAHI-SC2 induces cross-variant neutralizing antibodies, establishes key long-lived bone marrow-resident antibody-secreting cell populations, and generates robust Th1-biased polyfunctional T cell responses. AAHI-SC2 exhibits long-term thermostability, allowing it to be stored at room temperature or refrigerated conditions for at least 6 months—and possibly far longer—while still maintaining the ability to stimulate equivalent neutralizing antibody titers. This vaccine, soon to begin human testing in accelerated Phase I/II clinical trials, demonstrates excellent preclinical immunogenicity, allows for simpler manufacturing than mRNA vaccines currently in use, and avoids cold-chain requirements, enabling greater global vaccine access and distribution.

The two mRNA vaccines in widespread use are thought to be effective via induction of neutralizing antibody titers but rely on LNP-encapsulated nucleoside-modified RNA^46,47^. Our vaccine-elicited neutralizing antibody responses in mice well exceeded levels that have been predicted to be protective^48^ without the need for modified nucleosides as consistent with others^49,50^. While direct comparisons of our saRNA/NLC vaccine to the approved mRNA vaccines were not possible in this study due to limitations on access to these vaccines for research use, literature comparisons suggest that, acknowledging minor differences in the immunogenicity assays used, AAHI-SC2 humoral immunogenicity in C57BL/6J mice likely compares favorably with these mRNA vaccines^6,7^.

While some studies indicate that neutralizing antibody titers may be considered correlates of protection^51–55^, T cell responses have been shown to be critical for the control and clearance of infection, as well as for mediating protection against emerging variants and preserving long-term immunity^55–58^. T cell responses appear earlier than neutralizing antibodies following vaccination^59^, and the timing of their appearance correlates with the attainment of vaccine protective efficacy^3^. Although emerging variants of SARS-CoV-2 appear to be able to evade vaccine-induced humoral immune responses^60,61^, T cell immunity appears to be more durable in response to these variants^62,63^ likely because T cell epitopes are more highly conserved in emergent SARS-CoV-2 variants than the corresponding B cell epitopes^63^. Combined, the literature presents a strong case for the importance of vaccines that induce not only high levels of neutralizing antibodies but also strong and enduring T cell responses. The Moderna and Pfizer-BioNTech vaccines elicit strong levels of neutralizing antibodies^6,64^, but the T cell responses to these existing vaccines are less than robust in both preclinical and clinical studies^6,64^. AAHI-SC2 appears to elicit strong T cell responses in preclinical studies, possibly due to the combination of the adjuvanting properties of squalene and saRNA, and may provide a foundation for durable and long-term T cell immunity. Upcoming studies in non-human primates and clinical trials in humans should provide additional data to evaluate the immunogenicity, and particularly the T cell induction, of AAHI-SC2 for comparison to published data on mRNA-1273, BNT162b2, and other SARS-CoV-2 mRNA^6,7,65^ or saRNA^29,30,33–35^ vaccines in development.

Th1-biased vaccine immune responses are key for both durable protection and vaccine safety^66^. Th2 responses have been associated with immunopathology induced by vaccines^46,66,67^ or by natural infection with SARS-CoV-2^68^, while Th1-type responses are associated with durable vaccine immunity^69^ and a lack of observed immunopathology in COVID-19 patients^69,70^. It is unclear how widely Th2-type vaccine responses induced by coronavirus vaccines could lead to clinically meaningful immune complications, with no evidence of vaccine-associated enhanced respiratory disease or antibody-dependent enhancement of virus infection and disease reported in any human trials for SARS-CoV-1, SARS-CoV-2, or Middle East Respiratory Syndrome vaccines^71,72^. Regardless, the AAHI-SC2 vaccine clearly induces strong Th1-biased immunity suggesting a favorable immunogenicity profile for a SARS-CoV-2 vaccine.

In addition to its immunogenicity, thermostability of the AAHI-SC2 vaccine is critical for its potential utility. Several components of mRNA vaccines may affect their stability during storage: LNP formulation, modified nucleosides, GC content, RNA length, and non-coding regions^29,30^. While optimization of these properties has led to improved mRNA stability, stringent cold-chain requirements are still necessary for the Moderna and Pfizer-BioNTech vaccines^9,10^. These requirements were a monumental challenge in the early days of vaccine rollout and continue to be a major impediment to worldwide distribution^73^, particularly given the short stability window of the Moderna and Pfizer-BioNTech vaccines at room temperature (20–25 °C).

Additional RNA vaccines are currently under development with reported stability data, but these data are sparse and indicate limited, short-term room-temperature stability of days to a few weeks at best^13,45,65^. As these reports illustrate, maintenance of RNA integrity during storage and after administration is challenging. RNA molecules are more susceptible to degradation than DNA due to the presence of the 2’ hydroxyl group on the ribose backbone. RNAs must also be protected from degradation by ubiquitous ribonucleases. Lyophilization is a known strategy to stabilize RNA by itself for long-term storage;^74^ however, limited stability has been demonstrated for lyophilized RNA vaccines encapsulated in LNPs^17,18^. The complex structure of LNPs may make lyophilization difficult due to the potential for rupture of the lipid layers and/or payload leakage, particularly important due to the encapsulation of the RNA. In contrast, the NLC delivery vehicle, which has a structure more similar to an oil-in-water emulsion than an LNP, has previously demonstrated stability upon lyophilization^39^. While lyophilization does have the potential to introduce bottlenecks to the manufacturing process, it is an established technique employed by multiple licensed vaccines and biologics to promote the thermostability of biopharmaceutical products^75,76^. In pandemic response, a multi-faceted approach to global vaccine distribution is necessary, and lyophilization is an additional strategy that can be employed to reach the large number of people who have yet to receive a COVID-19 vaccine in countries with less-developed medical infrastructure^2^. Furthermore, the application of other drying techniques to RNA vaccines may retain the thermostability benefits of a dried product while minimizing potential scale-up challenges with lyophilization; such alternate drying technologies may be explored in future work. In this work, demonstrating 6 months of stability at ambient temperature and long-term stability at refrigerator temperatures, the AAHI-SC2 vaccine represents a potential major improvement in RNA vaccine stability and distribution given the current storage recommendations for licensed mRNA vaccines.

Besides its thermostability profile, AAHI-SC2 also offers several-manufacturing and supply-chain advantages relative to existing vaccines and vaccine candidates. NLC manufacturing relies on equipment already utilized in standard oil-in-water emulsion-based vaccine adjuvant production and materials that can be sourced from multiple different commercial vendors. In contrast to LNPs, which require encapsulation of the RNA during production, our vaccine technology allows for the NLC and RNA to be produced separately and complexed by simple mixing at any point up to just before administration. This approach greatly simplifies manufacturing, enables stockpiling of NLC for pandemic preparedness^39^, and allows for post-manufacturing substitution of saRNA species to address emerging viruses or viral variants of concern. The AAHI-SC2 vaccine also does not contain pseudouridine components or highly specialized proprietary lipids whose scarcity and proprietary nature present challenges for sourcing^22^.

## Summary

We present a potent SARS-CoV-2 saRNA vaccine that induces strong immune responses in mice and demonstrates establishment of long-lived antibody-secreting plasma cell populations. The potency of this vaccine in conjunction with its induction of broadly cross-variant neutralizing antibodies may allow this vaccine to provide effective protection against many SARS-CoV-2 strains. While this vaccine candidate is only now advancing into non-human primate studies, animal efficacy testing, and human trials, we believe it represents an important advance in RNA vaccine technology, particularly for rural areas and the developing world. Key advantages of this technology are its ease of manufacturing and thermostability, enabling lower costs and widespread distribution in lower-income countries without the need for an extreme cold chain. This vaccine is scheduled to advance into efficacy studies in non-human primates and an accelerated Phase I/II clinical trial. This thermostable saRNA/NLC vaccine technology is valuable not just for the current SARS-CoV-2 pandemic but also for future pandemic preparedness and responsiveness.

## Methods

### saRNA expression plasmid design, cloning, and production

Three saRNA plasmids each with a unique candidate SARS-CoV-2 spike open reading frame were created, along with a fourth saRNA plasmid expressing SEAP instead of a vaccine antigen as an appropriate vector control. The SARS-CoV-2 spike open reading frame sequence from GenBank MT246667.1 incorporating the additional D614G mutation was used as the “baseline” sequence, with additional modifications to create two additional vaccine candidates: D614G-2P represents the baseline sequence with a substitution of PP for KV at amino acid positions 987–988 and an addition of nine N-terminal codons from the reference genome encoding amino acid sequence MFLLTTKRT; D614G-2P-3Q refers to the baseline sequence with the diproline substitution and an additional substitution of QQAQ for RRAR at the furin cleavage site at amino acid positions 683–686. These sequences were then codon-optimized for mammalian (human) expression by Codex DNA (San Diego, CA) using a proprietary algorithm, synthesized by BioXp (Codex DNA), and inserted into AAHI’s backbone saRNA expression vector by Gibson cloning. The SEAP-expressing plasmid was created by a similar process to insert the SEAP expression sequence in place of the vaccine antigen. Plasmid sequences were all confirmed by Sanger sequencing. Template plasmids were amplified in E. coli and extracted using Qiagen (Germantown, MD) maxi- or gigaprep kits, followed by *Not*I linearization (New England Biolabs [NEB], Ipswich, MA). Linearized DNA was purified by Qiagen DNA purification kits.

### RNA manufacture

Vaccine saRNA was generated by T7 polymerase-mediated in vitro transcription (IVT) using *Not*I-linearized DNA plasmids as templates. An in-house optimized IVT protocol was used with commercially available rNTP mix (NEB) and commercially available T7 polymerase, RNase inhibitor, and pyrophosphatase enzymes (Aldevron, Fargo, ND). DNA plasmids were digested away (DNase I, Aldevron), and Cap 0 structures were added to the transcripts by treatment with guanylyltransferase (Aldevron), GTP, and S-adenosylmethionine (NEB). RNA was chromatographically purified using Capto Core 700 resin (GE Healthcare, Chicago, IL) followed by diafiltration and concentration through tangential flow filtration using a 750 kDa molecular weight cut-off modified polyethersulfone membrane (Repligen, Waltham, MA). The final bulk RNA contained 10 mM Tris-HCl pH 8. Terminal filtration of the saRNA material was done using a 0.22 µm PES filter, and the saRNA materials were stored at −80 °C until use/complexation. Agarose gel electrophoresis was used to characterize saRNA size and integrity. All gels that derive from the same experimental timepoint were processed in parallel. RNA concentration was quantified by UV absorbance (NanoDrop 1000) and RiboGreen assay (Thermo Fisher Scientific, Waltham, MA).

RNA for versions of saRNA with modified nucleosides was made with n^1^-methylpseudouridine-5’-triphosphate (TriLink BioTechnologies, San Diego, CA) substituted for UTP. The other three NTPs were used as normal, and production proceeded normally as described above.

### NLC manufacture

Nanostructured lipid carrier (NLC) formulation was produced as follows^38,39,44^. Trimyristin (Sigma-Aldrich, St. Louis, MO), squalene (SAFC Supply Solutions, St. Louis, MO), sorbitan monostearate (Spectrum Chemical Mfg. Corp., New Brunswick, NJ), and the cationic lipid DOTAP (Lipoid, Ludwigshafen, Germany) were mixed and heated at 60 °C in a bath sonicator to create the oil phase. Polysorbate 80 (MilliporeSigma, Burlington, MA) was separately diluted in 10 mM sodium citrate trihydrate and heated to 60 °C in a bath sonicator to create the aqueous phase. After dispersion of components in each phase, a high-shear mixer (Silverson Machines, East Longmeadow, MA) was used at ~5000 rpm to mix the oil and aqueous phases. Particle size of the mixture was then further decreased by high-pressure homogenization by processing at 30,000 psi for ten discrete passes using an M110P Microfluidizer (Microfluidics, Westwood, MA). The NLC product was then filtered through a 0.22 µm PES filter and stored at 2–8 °C until use.

### Vaccine complexing and characterization

Vaccine complexes for immediate in vivo injection were created by mixing aqueous RNA 1:1 by volume with NLC diluted in a buffer containing 10 mM sodium citrate and 20% w/v sucrose. All vaccines were prepared at nitrogen:phosphate (N:P) ratio of 15, representing the ratio of amine groups on the NLC DOTAP to phosphate groups on the RNA backbone. This produced a vaccine solution containing the intended dose of complexed saRNA/NLC in an isotonic 10% w/v sucrose, 5 mM sodium citrate solution (with small (<4 mM) amounts of Tris buffer present from the bulk RNA material). Vaccine was incubated on ice for 30 min after mixing to ensure complete complexing.

Hydrodynamic diameter (particle size) of the NLC and vaccine nanoparticles was determined using dynamic light scattering (Zetasizer Nano ZS, Malvern Panalytical, Malvern, UK) on triplicate 1:100 dilutions in nuclease-free water. Sizing was done in a disposable polystyrene cuvette using previously established parameters^39^. Zeta potential (particle charge) of the vaccine nanoparticles was also measured by dynamic light scattering in a disposable folded capillary cell using the same instrument with the same material and dispersant parameters.

Free and complexed RNA integrity and NLC-provided protection against RNases were evaluated by visualizing RNA integrity after agarose gel electrophoresis. RNA samples were diluted to 40 ng/μL RNA in nuclease-free water. For RNase treatment, this diluted RNA was incubated with RNase A (10 mg/mL, Thermo Scientific, Waltham, MA) at a mass ratio of 1:500 RNase:RNA for 30 min at room temperature, followed by incubation with proteinase K (~20 mg/mL, Thermo Scientific) at a mass ratio of 1:100 RNase A:proteinase K for 10 minutes at 55 °C. For complexed samples, RNA was extracted from complexes prior to gel electrophoresis by phenol:chloroform extraction. All RNA samples were mixed with glyoxal loading dye (Invitrogen, Waltham, MA) 1:1 by volume, incubated at 50 °C for 20 min, loaded on a denatured 1% agarose gel in NorthernMax-Gly running buffer (Invitrogen) alongside Millennium RNA Markers (Thermo Fisher Scientific), and run at 120 V for 45 minutes before imaging on a ChemiDoc MP Imaging System (Bio-Rad Laboratories, Hercules, CA). All gels that derive from the same experiment timepoint were processed in parallel.

### Vaccine lyophilization

Vaccine complexes intended for lyophilization and storage were prepared similarly to above by mixing aqueous RNA 1:1 by volume with NLC (in 10 mM sodium citrate) diluted in either 20% w/v sucrose only, 20% w/v sucrose and 5 mM sodium citrate, or 20% w/v sucrose with both 1 mM sodium citrate and 10 mM Tris. All vaccines with all excipient conditions contained approximately 2.6 mM Tris buffer from the bulk RNA material and were prepared at an N:P ratio of 15 and incubated on ice for 30 min after mixing to ensure complete complexing. After complexing, vaccine was lyophilized using a VirTis AdVantage 2.0 EL-85 (SP Industries, Warminster, PA) benchtop freeze dryer controlled by the microprocessor-based Wizard 2.0 software. The lyophilization cycle began with a freezing step at −50 °C, followed by primary drying at −30 °C and 50 mTorr, and finishing with secondary drying at 25 °C and 50 mTorr. When the cycle was complete, the samples were brought to atmospheric pressure, blanketed with high-purity nitrogen, and stoppered before being removed from the freeze-dryer chamber. At each timepoint, lyophilized samples were reconstituted using nuclease-free water to their original concentration.

### Mouse studies

All animal studies in this work were approved by the Bloodworks Northwest Research Institute’s Institutional Animal Care and Use Committee (IACUC) (Seattle, WA) and conducted at the Bloodworks Northwest Research Institute Animal Facility. All animal work was in compliance with all applicable sections of the Final Rules of the Animal Welfare Act regulations (9 CFR Parts 1, 2, and 3) and the *Guide for the Care and Use of Laboratory Animals, Eighth Edition*^77^. This work complied with all pertinent ethical regulations for animal testing and research.

C57BL/6J mice obtained from The Jackson Laboratory (Harbor, ME) were used for all animal studies in this work. Mice were between 6 and 8 weeks of age at study onset. Equal numbers of male and female mice were used for all studies, with the sole exception of the Fig. 6 stability study in which all female mice were used. Mice were immunized by intramuscular injection bilaterally in the rear quadriceps muscle (50 µL/leg, 100 µL total). Survival blood samples were taken by retro-orbital bleed; terminal blood samples were taken by cardiac puncture.

### Serum IgG, IgG1, and IgG2a titers by ELISA

SARS-CoV-2 spike protein-binding IgG antibodies in mouse serum were measured by ELISA. Plates (384-well high-binding plates, Corning, Corning, NY) were coated with 1 µg/mL of Recombinant SARS-CoV-2 Spike His Protein, Carrier Free, (R&D Systems, Minneapolis, MN; #10549-CV) in phosphate-buffered saline (PBS) and incubated overnight at 4 °C. The coating solution was removed, and blocking buffer (2% dry milk, 0.05% Tween 20, and 1% goat serum) was applied for at least 1 h. On a separate, low-binding plate, each sample was diluted 1:40 and then serially 1:2 to create a 14-point dilution curve for each sample. Naïve mouse serum, used as the negative control, was diluted identically to the samples. A SARS-CoV-2 neutralizing monoclonal antibody (mAb; GenScript, Piscataway, NJ; #A02057), was used as a positive control at a known starting concentration of 3.2 ng/µL followed by serial 1:2 dilutions similarly to each sample and negative control. SARS-CoV-2 spike protein-coated and blocked assay plates were washed, and serially diluted samples were then transferred onto the coated plates followed by a 1-h incubation. Plates were then washed, and spike protein-bound antibodies were detected using an Anti-Mouse IgG (Fc Specific)-Alkaline Phosphatase antibody (Sigma-Aldrich, #A2429) at a 1:4000 dilution in blocking buffer. Plates were washed and then developed using phosphatase substrate (Sigma-Aldrich, #S0942) tablets dissolved in diethanolamine substrate buffer (Fisher Scientific, Waltham, MA; #PI34064) at a final concentration of 1 mg/mL. After a 30-minute development, plates were read spectrophotometrically at 405 nm. A 4-point logistic curve was used to fit the antibody standard titration curve. Sample concentrations were interpolated off the linear region of each sample dilution curve using the standard curve for absolute quantification of antibody titers. For Supplementary Fig. 4 only, antibody level was quantified by endpoint titer, with the endpoint titer defined as the dilution at which each sample dilution curve rises to above 3 standard deviations above assay background.

For IgG1 and IgG2a isotype-specific ELISAs, the identical plate coating and blocking procedures were conducted. For the IgG1 assay, the standard curve was run using an IgG1 SARS-CoV-2 neutralizing antibody (GenScript #A02055) or an IgG2a SARS-CoV-2 neutralizing antibody (GenScript #BS-M0220) for full quantification. Sample dilution and incubation were identical to the total IgG curve, and plates were probed with IgG1- and IgG2a-specific secondary alkaline phosphatase-conjugated detection antibodies prior to development, reading, and quantification as described above.

### Pseudovirus neutralization assay

The SARS-CoV-2 pseudovirus-neutralizing antibody titers of immunized mouse sera were measured by pseudovirus neutralization assays using procedures adapted from Crawford et al.^78^. Lentiviral SARS-CoV-2 spike protein pseudotyped particles were prepared by following the Bioland Scientific (Paramount, CA) BioT plasmid transfection protocol. Briefly, HEK-293 cells (American Type Culture Collection, Manassas, VA; #CRL-3216) were plated at 4 × 10^5^ cells/mL in six-well plates 18–24 hours before the assay to achieve 50–70% confluency at assay start. Cellular growth medium was then replaced with serum-free Gibco Dulbecco’s Modified Eagle Medium (DMEM) with GlutaMAX immediately prior to transfection. The HEK-293 cells were co-transfected with several plasmids: a plasmid containing a lentiviral backbone expressing luciferase and ZsGreen (BEI Resources, Manassas, VA; #NR-52516), plasmids containing lentiviral helper genes (BEI Resources; #NR-52517, NR-52518, and NR-52519), and a plasmid expressing a delta19 cytoplasmic tail-truncated SARS-CoV-2 spike (Wuhan strain, B.1.1.7, and B.1.351 plasmids from Jesse Bloom, Fred Hutchinson Cancer Center, Seattle, WA; and B.1.617.2 plasmid from Thomas Peacock, Imperial College London, UK). BioT transfection reagent (Bioland Scientific) was used to mediate the co-transfection. The assay plates were incubated for 24 h at standard cell culture conditions (37 °C and 5% CO_2_). Then the serum-free media was aspirated off, and fresh growth medium (Gibco DMEM with GlutaMAX and 10% fetal bovine serum [FBS]) was added to the plates before returning them to the incubator for an additional 48 h. Pseudovirus stocks were collected by harvesting the supernatants from the plates, filtering them through a 0.2 μm PES filter (Thermo Scientific), and freezing at −80 ^o^C until titration and use.

Mouse serum samples were diluted 1:10 in medium (Gibco DMEM with GlutaMAX and 10% FBS) and then serially diluted 1:2 for 11 total dilutions in 96-well V-bottom plates. Polybrene (Sigma-Aldrich) was then added at a concentration of 5 µg/mL to every well on the plate, and pseudovirus, diluted to a titer of 1 × 10^8^ total integrated intensity units/mL as per titers determined independently for each pseudovirus batch, was added 1:1 to the diluted serum samples. The plates were incubated for 1 h at 37 °C and 5% CO_2_. Serum-virus mix was then added in duplicate to Human Angiotensin-Converting Enzyme 2-expressing HEK-293 cells (BEI Resources, #NR-52511) seeded at 4 × 10^5^ cells/mL on a 96-well flat-bottom plate and incubated at 37 °C and 5% CO_2_ for 72 hours. To determine 50% inhibitory concentration (IC_50_) values, plates were scanned on a high-content fluorescent imager (ImageXpress Pico Automated Cell Imaging System, Molecular Devices, San Jose, CA) for ZsGreen expression. Total integrated intensity per well was used to calculate the percent of pseudovirus inhibition in each well. Neutralization data for each sample were fit with a four-parameter sigmoidal curve that was used to interpolate IC_50_ values.

A Wuhan-strain pseudoneutralization test using the World Health Organization (WHO) standard for neutralization assays with an official IC_50_ of 1000 resulted in an IC_50_ of 7800 using our assay, suggesting that our test is more sensitive than the WHO assay but may overreport IC_50_ values—by less than one log_10_—an effect likely most pronounced for strongly neutralizing samples.

### Splenocyte harvest, intracellular cytokine staining, and flow cytometry

Spleens were dissociated in 4 mL of RPMI medium by manual maceration through a cell strainer using the end of a syringe plunger. Homogenized samples were briefly centrifuged at 400 × *g* (15–20 s) to pellet fat cells. Samples were then carefully resuspended and fat clumps were removed by pipette. The supernatants containing lymphocytes were transferred to 5-mL mesh-cap tubes to strain out any remaining tissue debris or were lysed with ammonium-chloride-potassium buffer and washed. Cell counts for each sample were obtained on a Guava easyCyte (Luminex, Austin, TX). Each spleen sample was seeded in 96-well round-bottom plates at 1–2 × 10^6^ cells per well in RPMI medium containing 10% FBS, 50 μM beta-mercaptoethanol, CD28 costimulatory antibody (0.4 μL/test, BD Biosciences #553294), and brefeldin A. Cells were stimulated with one of three stimulation treatments: 0.0475% dimethyl sulphoxide (DMSO) as a negative stimulation control, 0.2 μg/well (1 μg/mL) per peptide of spike peptide pool (JPT Peptide Technologies, Berlin, Germany; #PM-WCPV-S-1) in an equivalent amount of DMSO, or 10 μg/well of phorbol myristate acetate (PMA)/ionomycin solution. After 6 h of incubation at 37 °C with 5% CO_2_, plates were centrifuged at 400 × *g* for 3 min, the supernatants were removed by pipetting, and cells were resuspended in PBS. Plates were centrifuged, the supernatants were removed, and cells were stained for flow cytometry. Splenocytes were stained for viability with Zombie Green (BioLegend, San Diego, CA) in 50 μL of PBS, and then Fc receptors were blocked with CD16/CD32 antibody (0.25 μL/test, Invitrogen #14–0161–86). Cells were then surface stained with fluorochrome-labeled mAbs specific for mouse CD4 (0.3 μL/test, PerCP-Cy5.5, eBioscience #45–0042–82), CD8 (2 μL/test, BV510, BD Biosciences #563068), CD44 (0.2 μL/test, APC-Cy7, BD Biosciences #560568), and CD107a (2 μL/test, APC, BioLegend #121614) in 50 μL of staining buffer (PBS with 0.5% bovine serum albumin and 0.1% sodium azide). Cells were washed twice, permeabilized using the Fixation/Permeabilization Kit (BD Biosciences, Franklin Lakes, NJ), and stained with fluorochrome-labeled mAbs specific for mouse TNFα (2 μL/test, BV421, BioLegend #506327), IL-2 (0.4 μL/test, PE-Cy5, BioLegend #503824), IFNγ (0.2 μL/test, PE-Cy7, Invitrogen #25731182), IL-5 (0.7 μL/test, PE, eBioscience #12–7052–82), IL-10 (2 μL/test, BV711, BD Biosciences #564081), and IL-17A (2 μL/test, AF700, BD Biosciences #560820). After two washes in staining buffer, cells were resuspended in 100 μL of staining buffer and analyzed on an LSRFortessa flow cytometer (BD Biosciences). After initial gating for live CD4^+^ or CD8^+^ lymphocytes, cells were gated for cytokine positivity. Quality of the response was determined by gating on cells that were double or triple positive for these markers. Cells triple positive for TNFα, IL-2, and IFNγ were considered activated polyfunctional T cells.

### T-cell ELISpot assay

ELISpot plates (MilliporeSigma) were coated with either IFNγ (BD Biosciences, #51–2525KZ), IL-17A (Invitrogen, #88–7371–88), or IL-5 (BD Biosciences, #51–1805KZ) capture antibodies at a 1:200 dilution in Dulbecco’s PBS (DPBS; Gibco). After overnight incubation at 4 ^o^C, plates were washed and then blocked with complete RPMI (cRPMI) medium for at least 2 h. Splenocytes harvested as described above were plated at 2 × 10^5^ cells per well. A subset of each sample was stimulated with PepMix SARS-CoV-2 (JPT Peptide Technologies, #PM-WCPV-S-1) at a final concentration of 1 µg/mL. Plates were then incubated at 37 °C and 5% CO2 for 48 h. After a wash with PBS with 0.1% Tween 20, 100 µL of detection antibody (IFNγ, BD Biosciences, #51–1818KA; IL-17A, Invitrogen, #88–7371–88; and IL-5, BD Biosciences, #51–1806KZ) was added at a 1:250 dilution in ELISpot diluent (eBioscience) overnight at 4 °C. Plates were washed and developed with Vector NovaRED Substrate Peroxidase (Vector Laboratories, Burlingame, CA; #SK-4800) for 15 min. The reaction was stopped by washing the plates with deionized water, and plates were left to dry in the dark. Spots were counted and data were analyzed using ImmunoSpot 7 software (Cellular Technology Limited, Cleveland, OH).

### Bone marrow IgA and IgG antibody-secreting cell ELISpot assays

Presence of antibody-secreting bone marrow-resident cells was measured by ELISpot. MultiScreen IP Filter plates (0.45 µm, MilliporeSigma) were treated with 15 µL of 35% ethanol for 30 s. After a wash with PBS, plates were coated with 100 µL of Recombinant SARS-CoV-2 Spike His Protein, Carrier Free, (R&D Systems, #10549-CV-100) at a concentration of 2 µg/mL diluted in DPBS (Gibco). Plates were incubated overnight at 4 °C, washed with PBS with 0.1% Tween 20, and blocked with cRPMI medium for at least 2 h.

To prepare bone marrow, both femurs were removed from each mouse and inserted into a snipped-end 0.6 mL tube (Eppendorf, Hamburg, Germany) inserted into a 1.5 mL Eppendorf tube containing 1 mL of cRPMI medium. Femurs were centrifuged for 15 s at 840 × *g*, and supernatant was discarded. The cell pellets were briefly vortexed and resuspended in 200 µg of RBC lysis buffer (pH 7.1–7.4) (Invitrogen), and they were incubated on ice for 30 s. After addition of 800 μL of cRPMI medium, cells were centrifuged 5 min at 400 × *g*, and supernatant was decanted. Cells were resuspended in 1 mL of cRPMI medium, counted, and transferred to prepared filter plates described above at 1 million cells per well followed by a threefold dilution across five adjacent wells.

After a 3-h incubation, plates were washed with PBS with 0.1% Tween 20, and secondary antibody (Goat Anti-Mouse IgG-HRP or IgA-HRP [SouthernBiotech, Birmingham, AL; #1030–05 and #1040–05]) was added at a 1:1000 dilution in PBS with 0.1% Tween and 5% FBS overnight at 4 °C. Plates were then washed three times in PBS with 0.1% Tween 20 and two times in PBS. For development, 100 µL of Vector NovaRED Substrate Peroxidase (Vector Laboratories, #SK-4800) was applied for 7 min. The reaction was stopped by rinsing plates with distilled water for 2 min, and plates were dried in the dark. Spots were counted and data were analyzed using ImmunoSpot software (Cellular Technology Limited).

### Statistical analyses

Log-normalized IgG levels for vaccine storage analysis were analyzed by one-way ANOVA at each timepoint followed by Dunnett’s multiple comparison test comparing each stored sample to the freshly complexed positive control at each timepoint. Log-normalized IgG titers to assess prime and boost at multiple doses or with different vaccine constructs at prime and boost were assessed by mixed-effects analysis, or two-way ANOVA if there were no gaps in the data, both with and without multiple comparisons as needed. Log-normalized pseudovirus variant neutralization was compared using mixed-effects analysis or two-way ANOVA with multiple comparison correction. Bone marrow ELISpots were analyzed with one-way ANOVA with multiple comparisons. All statistical analyses were conducted using Prism 9 (GraphPad Software, San Diego, CA).

### Reporting summary

Further information on research design is available in the Nature Research Reporting Summary linked to this article.

## Supplementary information

Supplemental Material

Reporting Summary

## Data Availability

The datasets generated during the current study are available from the corresponding author (Emily A. Voigt, emily.voigt@aahi.org) upon request.
