# Supplementary material for: A self-amplifying RNA vaccine against COVID-19 with long-term room-temperature stability
Source: NPJ Vaccines. 2022 Nov 2;7:136. doi: 10.1038/s41541-022-00549-y (PMC9628444; doi:10.1038/s41541-022-00549-y)
Supplement: Supplementary file 1 — Supplemental Material [file 41541_2022_549_MOESM1_ESM.pdf]

SUPPLEMENTARY FIGURES

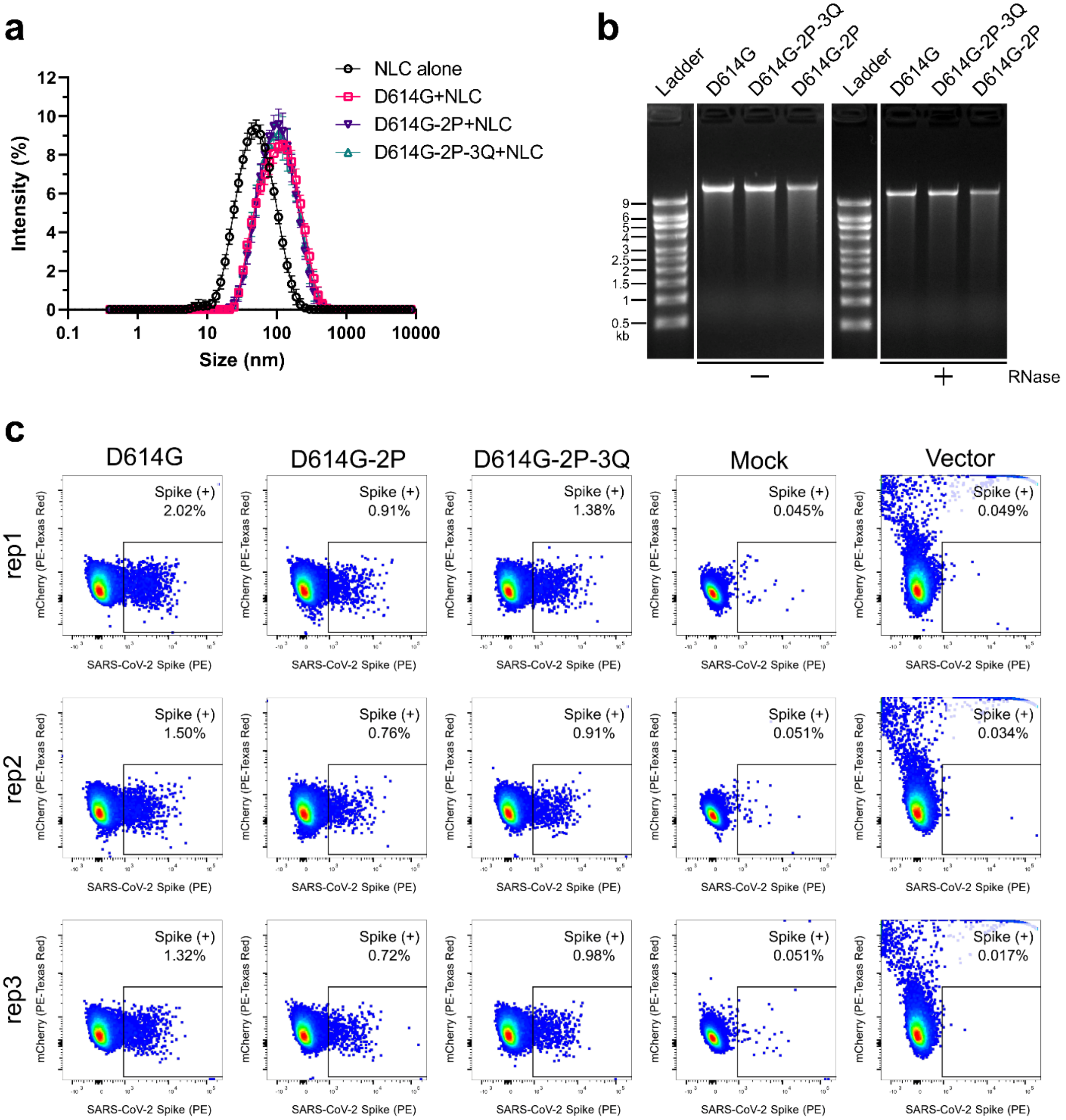

Supplementary Figure 1. Characterization of SARS-CoV-2 saRNA/NLC vaccine constructs D614G (baseline), D614G-2P, and D614G-2P-3Q (AAHI-SC2). a) Size distribution of NLC particles alone and saRNA/NLC vaccine particles after complexing with each saRNA construct, showing mean and standard deviation for *n* = 3 replicate measurements for each sample. **b**) RNA integrity by agarose gel electrophoresis for each saRNA construct complexed to NLC particles (– RNase) and integrity of each saRNA complex after treatment with RNase (+ RNase). Panels for each RNase treatment were derived from the same gel. See Supplementary Figure 9 for original unprocessed gel images. **c**) Flow cytometric detection of SARS-CoV-2 spike expression by HEK-293 T cells after *in vitro* transfection of 500 µg

saRNA/NLC per well of a cell-seeded 12-well plate. Cells were harvested 24 hours post-transfection and stained with a Cy3-conjugated rabbit polyclonal antibody to SARS-CoV-2 spike (Abcam, Cambridge, UK; #AB272504). Flow cytometry data for the three saRNA/NLC vaccine constructs and two controls (mock and mCherry reporter vector) include three biological transfection replicates. Cells transfected with the mCherry reporter vector/NLC demonstrate expression of mCherry but no background SARS-CoV-2 staining as expected.

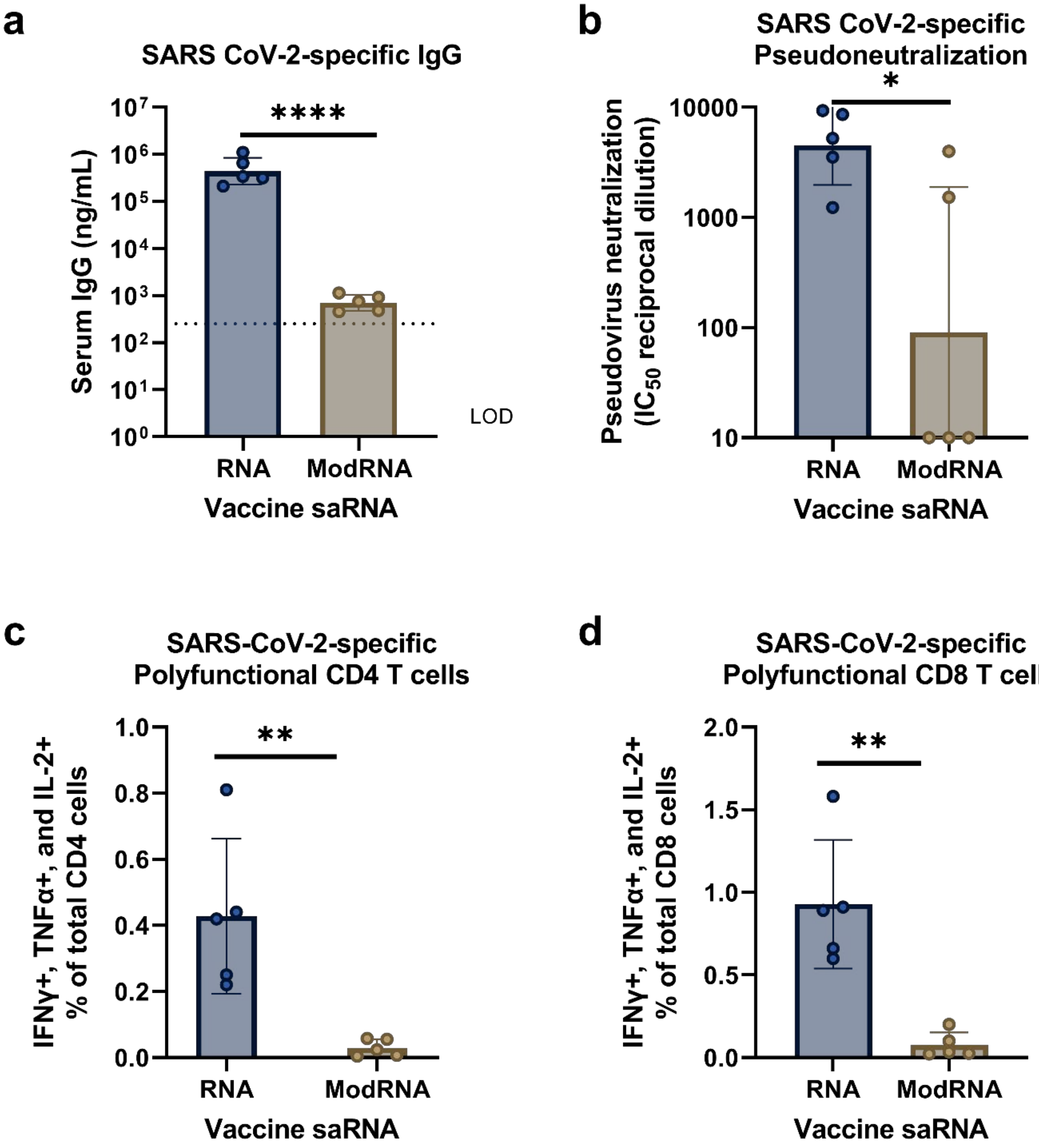

Supplementary Figure 2. Impact of modified nucleosides on saRNA/NLC vaccine immunogenicity. saRNA constructs for D614G-2P-3Q (AAHI-SC2) were produced with modified nucleosides (modRNA) or conventionally (RNA). Mice were primed and boosted with these constructs using 5 µg doses (n = 5/group), and serum and systemic immune responses were analyzed 3 weeks post-boost. **a**) Serum SARS-CoV-2 spike protein-binding IgG. LOD = limit of detection. **b**) Serum SARS-CoV-2 neutralizing antibodies against the original Wuhan strain. **c-d**) Spleen-resident polyfunctional T cells (IFNγ+, IL-2+, and TNFα+ CD4 or CD8 T cells). Results show geometric mean and geometric standard deviation (panels **a** and **b**) or mean and standard deviation (panels **c** and **d**). Statistics evaluated using an unpaired *t*-test. *$p < 0.05$, **$p < 0.01$, ****$p < 0.0001$.

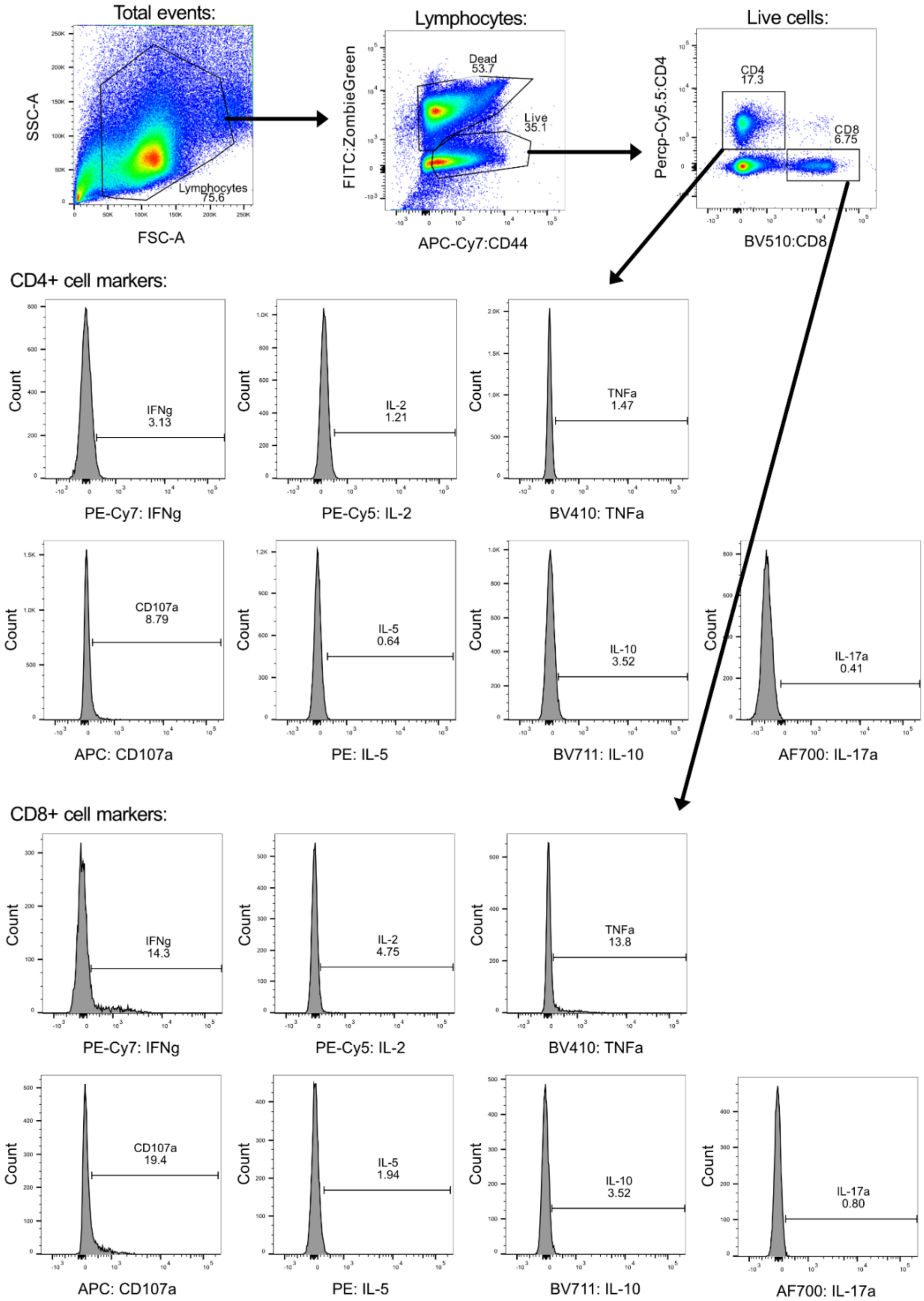

Supplementary Figure 3. Flow cytometry gating strategies for immunogenicity experiments in Figure 5. Isolated splenocytes were stained and analyzed via flow cytometry. Total splenocytes were gated first on total lymphocytes. The live cell subset was gated as FITC- and CD44+. Then individual gates were drawn for CD4 (CD4+CD8-) and CD8 cells (CD4-CD8+). Within each of these subsets, specific cytokines were gated using histograms, including IFNg, IL-2, TNFa, CD107a, IL-5, IL-10, and IL-17a.

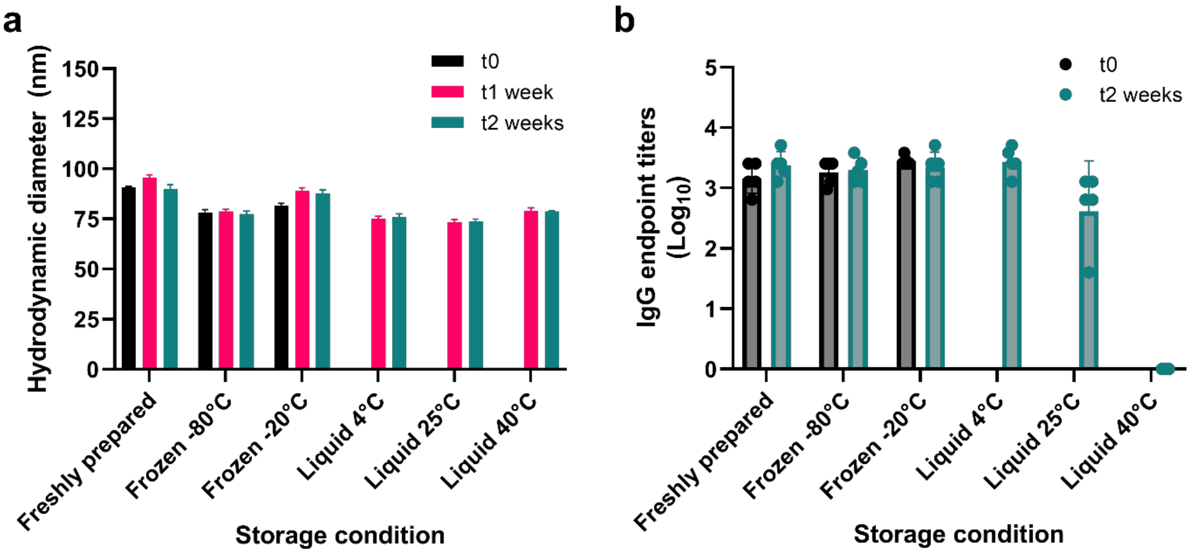

Supplementary Figure 4. Stability of the liquid SARS-CoV-2 saRNA/NLC vaccine (AAHI-SC2) after 2 weeks of storage at different temperatures. a) Mean hydrodynamic (Z-average) diameter of the vaccine complex prior to or after storage at the given conditions with error bars indicating the standard deviation (SD) of n = 3 replicate measurements for each condition at each timepoint. **b**) Serum SARS-CoV-2 spike protein-binding IgG induced in C57BL/6 mice by a single 10 μg dose of vaccine after storage at the indicated conditions. n = 5 mice/group. Serum samples were taken 2 weeks after vaccine injection and analyzed by ELISA. Results show geometric mean endpoint titer with error bars indicating the geometric SD.

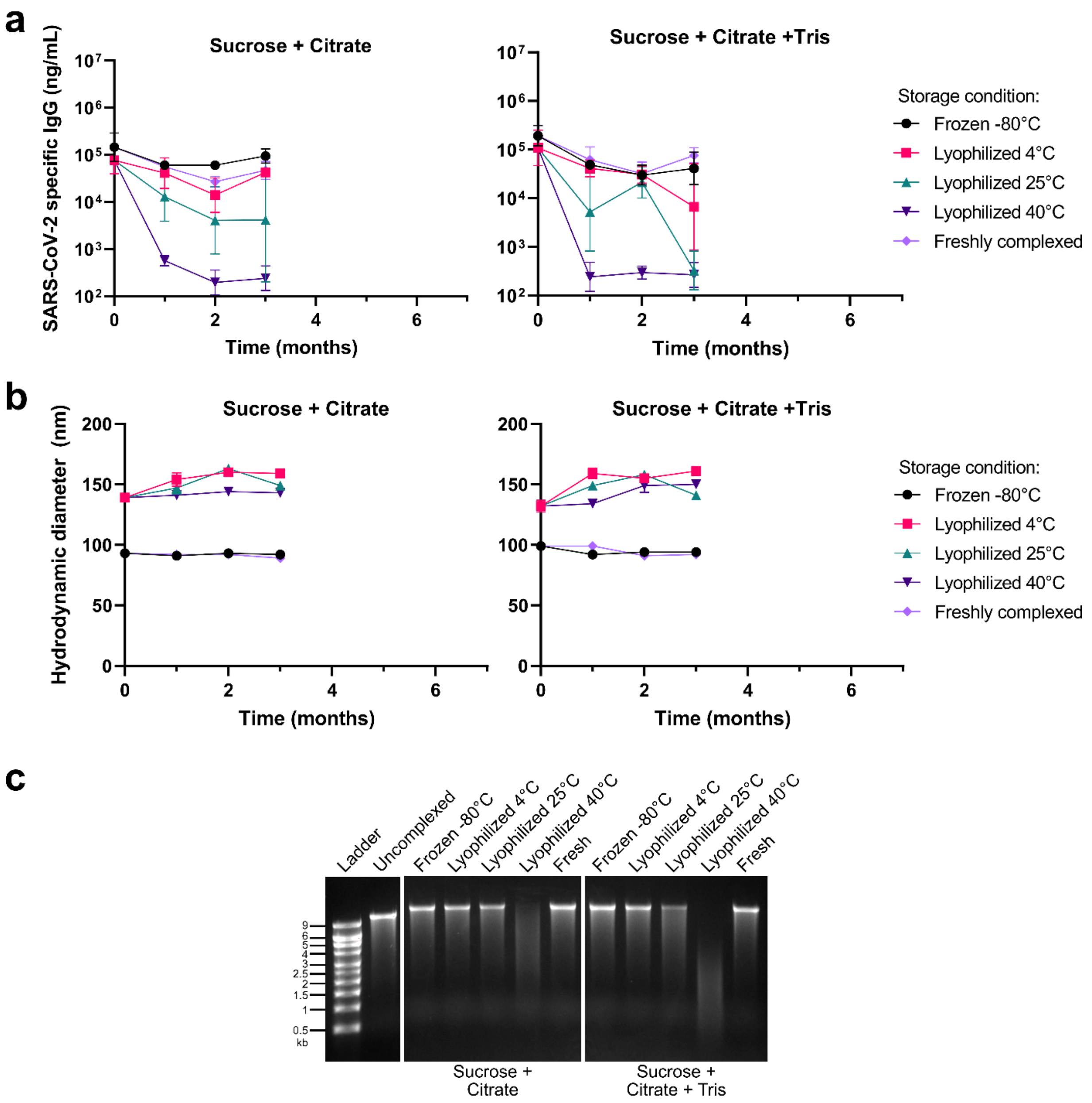

Supplementary Figure 5. Stability of the lyophilized SARS-CoV-2 saRNA/NLC vaccine (AAHI-SC2) in alternative excipient backgrounds after 3 months of storage at different temperatures. a) Serum SARS-CoV-2 spike protein-binding IgG after vaccine storage under the indicated conditions. *n* = 5 mice/group. Antibody plots show geometric mean and geometric standard deviation (SD). **b**) Hydrodynamic (Z-average) diameter of the vaccine complex. Results show mean and SD of *n* = 3 replicate measurements for each condition at each timepoint. **c**) Integrity of vaccine RNA after 3 months of storage under the indicated conditions. The sucrose + citrate formulation contained 20% w/v sucrose and 5 mM sodium citrate; the sucrose + citrate + Tris formulation contained 20% sucrose, 1 mM sodium citrate, and 10 mM Tris. Both formulations contained approximately 2.6 mM Tris buffer from the bulk RNA material. Panels were derived from the same gel. See Supplementary Figure 10 for the original unprocessed gel image.

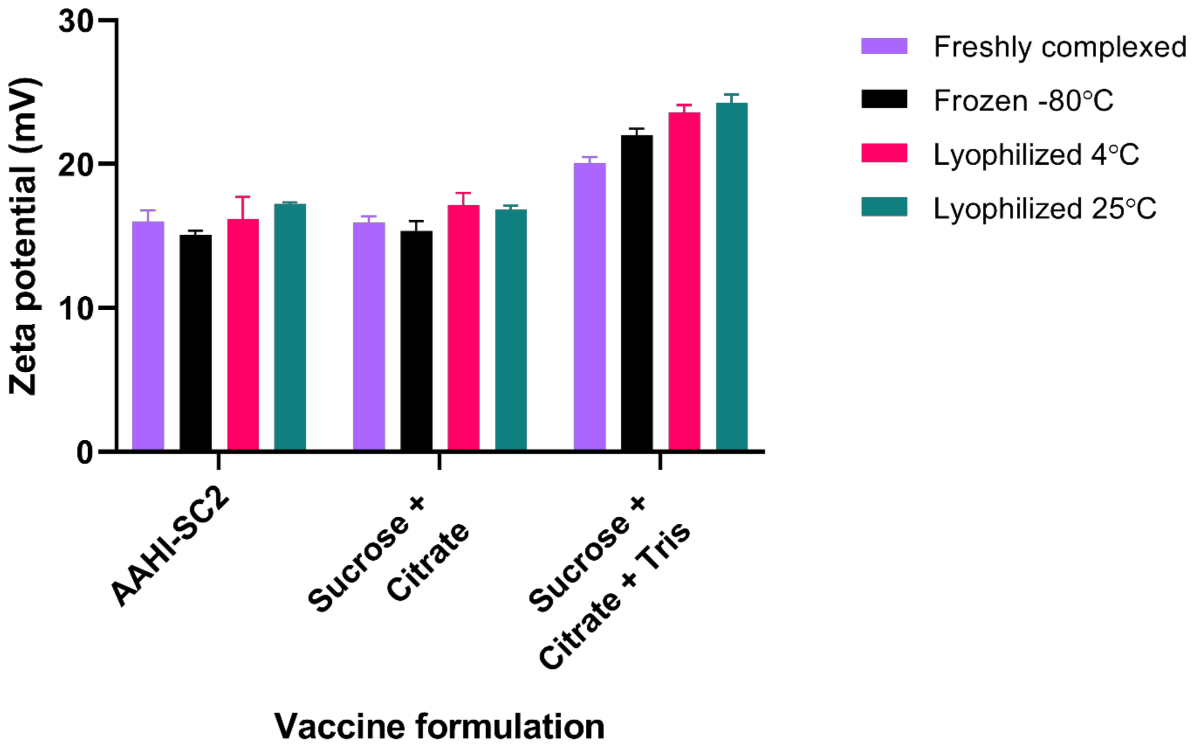

Supplementary Figure 6. Particle surface charge (zeta potential) of SARS-CoV-2 saRNA/NLC vaccine formulations after 12 months of storage under different conditions. Zeta potential was measured by dynamic light scattering with the results showing mean and standard deviation of n = 3 replicate measurements for each condition. Lyophilized samples were reconstituted with RNase-free water prior to measurement. The AAHI-SC2 saRNA/NLC vaccine contained 20% w/v sucrose and 2 mM sodium citrate. The sucrose + citrate alternative formulation contained 20% w/v sucrose and 5 mM sodium citrate. The sucrose + citrate + Tris alternative formulation contained 20% sucrose, 1 mM sodium citrate, and 10 mM Tris. All formulations contained approximately 2.6 mM Tris buffer from the bulk RNA material. Freshly complexed positive controls used saRNA that had been stored at -80°C in 10 mM Tris buffer, pH 8, and NLC stored at 4°C in 10 mM sodium citrate.

a

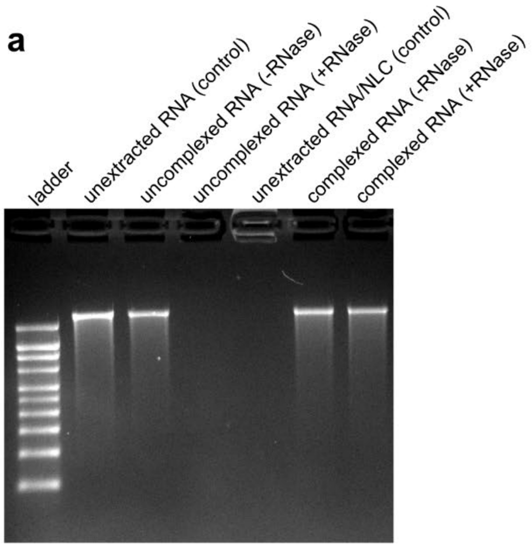

b

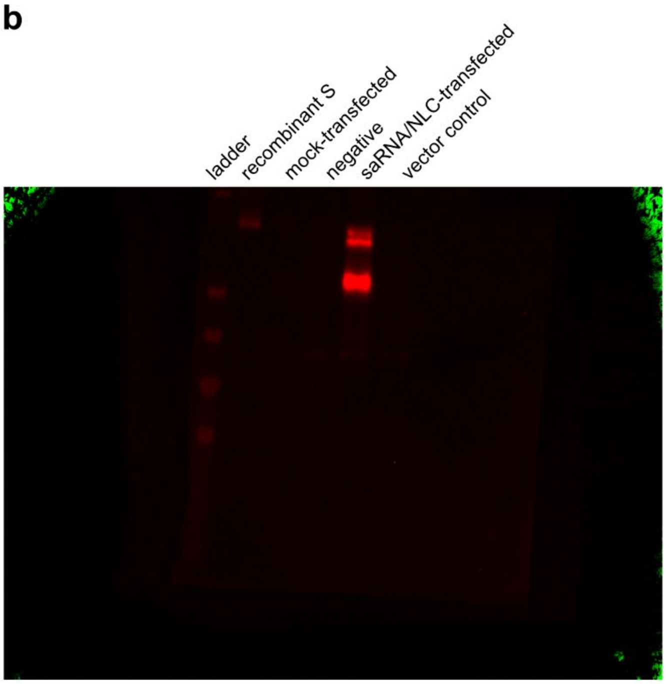

Supplementary Figure 7. Original unprocessed gel and blot images for Figure 1. **a**) Original gel image for Figure 1d showing RNA integrity for saRNA that is complexed to NLC and then extracted to run on the gel (as described in the Methods), uncomplexed, or unextracted as a control. Some samples were exposed to RNase (+RNase), and others were not (-RNase). **b**) Original western blot for Figure 1e showing SARS-CoV-2 spike (S) protein expression in saRNA/NLC vaccine-transfected HEK-293 cells.

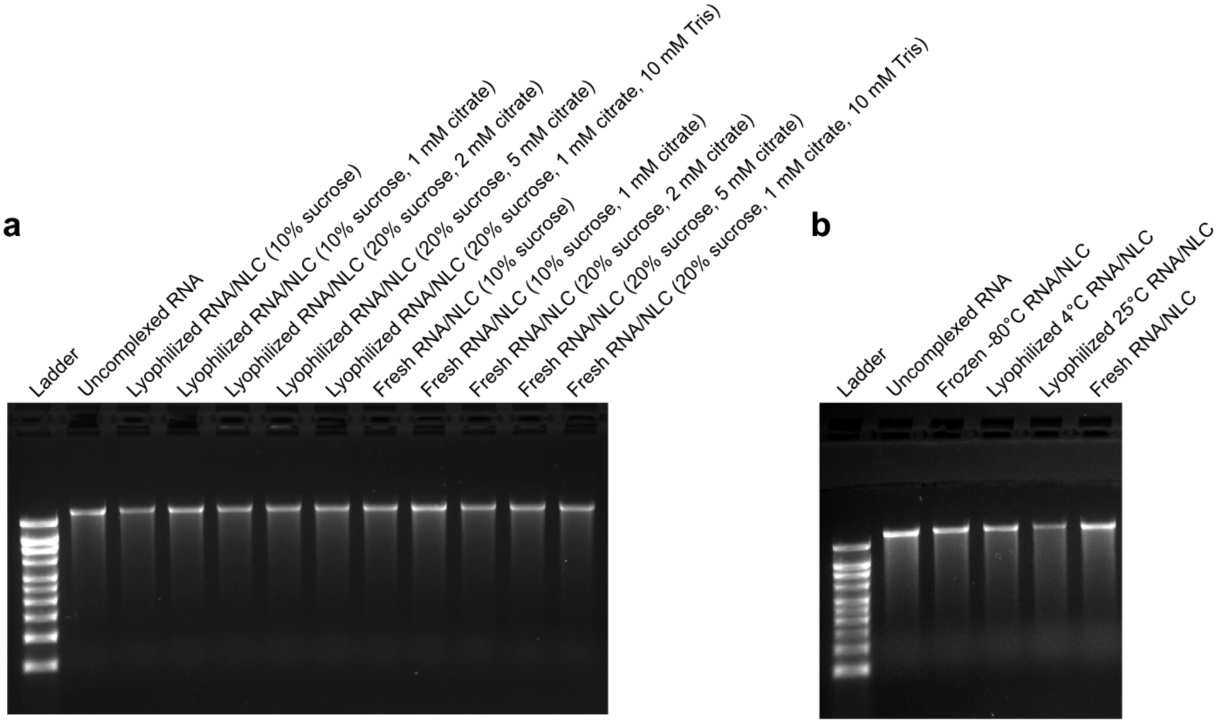

Supplementary Figure 8. Original unprocessed gel images for Figure 6 showing integrity of vaccine RNA at 0 and 10 months of storage at the indicated temperatures. “Uncomplexed RNA” refers to saRNA alone that had been stored at -80°C for the indicated length of time. **a**) Vaccine RNA integrity at 0 months of storage using different excipient backgrounds. The selected AAHI-SC2 excipient background (as shown in Figure 6) is represented by 20% sucrose, 2 mM citrate. **b**) Vaccine RNA integrity at 10 months of storage from the selected 20% sucrose, 2 mM citrate excipient background.

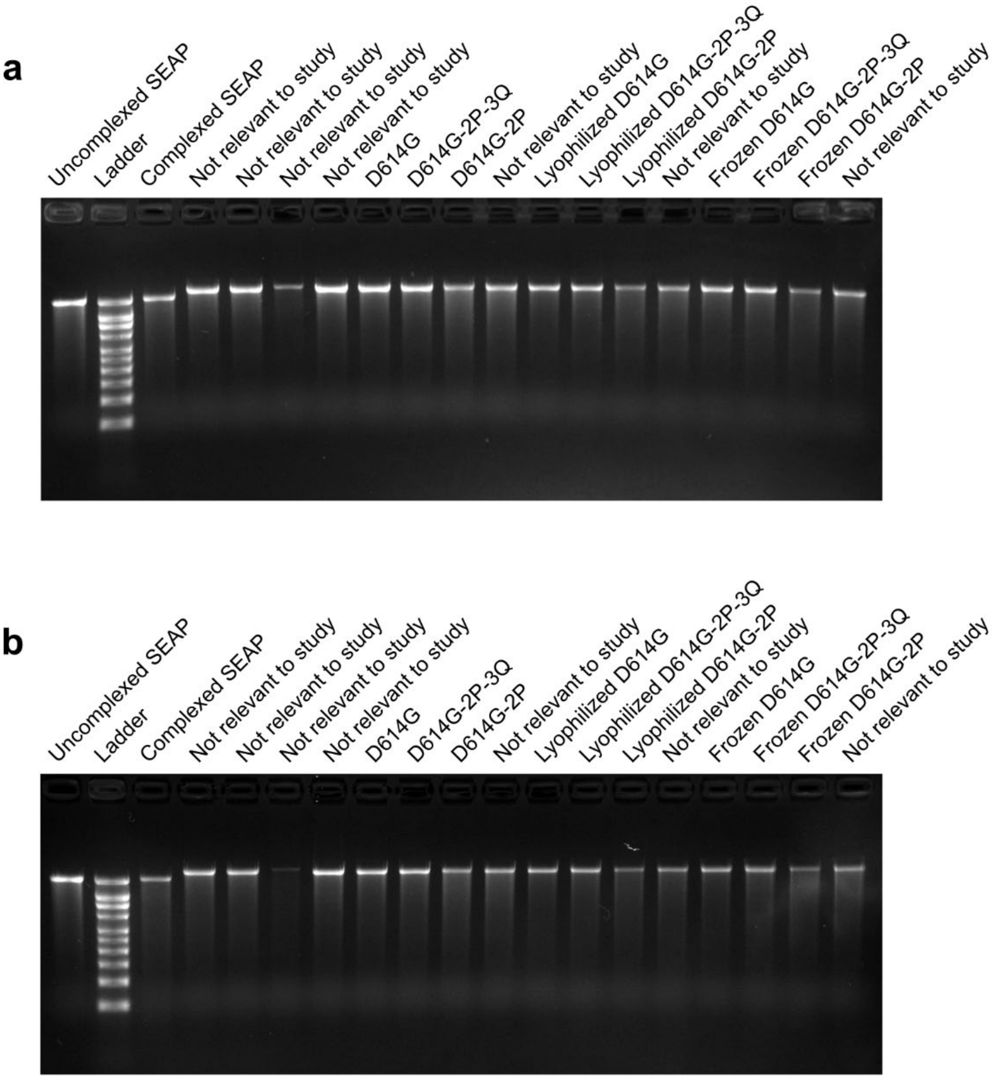

Supplementary Figure 9. Original unprocessed gel images for Supplementary Figure 1 showing RNA integrity of different saRNA constructs complexed to NLC using SEAP as a control. All samples were freshly prepared unless noted as lyophilized or frozen. Several samples on original gel were not relevant to this study and are indicated as such. **a**) RNA integrity without RNase treatment. **b**) RNA integrity after RNase treatment.

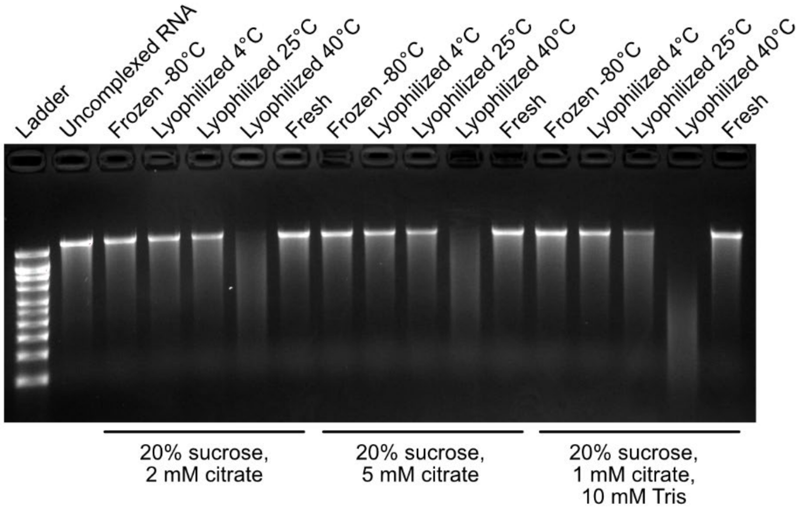

Supplementary Figure 10. Original unprocessed gel image for Supplementary Figure 5 showing Integrity of AAHI-SC2 vaccine RNA after 3 months of storage under the indicated conditions using different excipient backgrounds compared to freshly prepared vaccine.

SUPPLEMENTARY TABLES

Supplementary Table 1. Physicochemical stability of NLCs using DOTAP from different vendors, stored at 2-8°C

	Time point	NLC particle diameter (Z-ave, nm)	NLC size polydispersity index (PDI)	NLC visual appearance	NLC pH	NLC DOTAP content (mg/mL)	NLC squalene content (mg/mL)
**Expected range**	--	40 +/- 20	for information only	White or slightly yellow, translucent, single phase	5.0-6.5	30.0 +/- 9.0	37.5 +/- 7.5
**DOTAP vendor 1**	T = 0	38.3 +/- 0.8	0.19 +/- 0.01	White or slightly yellow, translucent, single phase	5.8	25.1 +/- 1.0	32.9 +/- 0.3
	T = 3 mon	42.0 +/- 0.3	0.26 +/- 0.02	White or slightly yellow, translucent, single phase	5.6	25.8 +/- 0.3	33.6 +/- 0.3
**DOTAP vendor 2**	T = 0	46.9 +/- 1.1	0.28 +/- 0.00	White or slightly yellow, translucent, single phase	5.7	24.2 +/- 1.8	31.0 +/- 1.4
	T = 3 mon	48.8 +/- 1.0	0.28 +/- 0.01	White or slightly yellow, translucent, single phase	5.6	26.8 +/- 0.9	35.1 +/- 0.6
**DOTAP vendor 3**	T = 0	41.2 +/- 0.2	0.16 +/- 0.00	White or slightly yellow, translucent, single phase	5.8	26.9 +/- 1.0	35.3 +/- 1.4
	T = 3 mon	43.1 +/- 0.4	0.18 +/- 0.01	White or slightly yellow, translucent, single phase	5.6	27.1 +/- 0.5	36.2 +/- 0.1

**DOTAP vendor 4**	T = 0	39.8 +/- 0.4	0.18 +/- 0.01	White or slightly yellow, translucent, single phase	5.8	31.8 +/- 0.1	37.1 +/- 0.2
	T = 3 mon	40.7 +/- 0.4	0.17 +/- 0.01	White or slightly yellow, translucent, single phase	6.2	32.6 +/- 0.1	37.7 +/- 0.3
