# Supplementary material for: A self-amplifying RNA vaccine against COVID-19 with long-term room-temperature stability
Source: NPJ Vaccines. 2022 Nov 2;7:136. doi: 10.1038/s41541-022-00549-y (PMC9628444; doi:10.1038/s41541-022-00549-y)
Supplement: Supplementary file 2 — Reporting Summary [file 41541_2022_549_MOESM2_ESM.pdf]

nature portfolio

Corresponding author(s): Emily Voigt

Last updated by author(s): Sep 13, 2022

Reporting Summary

Nature Portfolio wishes to improve the reproducibility of the work that we publish. This form provides structure for consistency and transparency in reporting. For further information on Nature Portfolio policies, see our Editorial Policies and the Editorial Policy Checklist.

Statistics

For all statistical analyses, confirm that the following items are present in the figure legend, table legend, main text, or Methods section.

n/a	Confirmed	
☐	☒	The exact sample size (*n*) for each experimental group/condition, given as a discrete number and unit of measurement
☐	☒	A statement on whether measurements were taken from distinct samples or whether the same sample was measured repeatedly
☐	☒	The statistical test(s) used AND whether they are one- or two-sided<br>*Only common tests should be described solely by name; describe more complex techniques in the Methods section.*
☒	☐	A description of all covariates tested
☐	☒	A description of any assumptions or corrections, such as tests of normality and adjustment for multiple comparisons
☐	☒	A full description of the statistical parameters including central tendency (e.g. means) or other basic estimates (e.g. regression coefficient) AND variation (e.g. standard deviation) or associated estimates of uncertainty (e.g. confidence intervals)
☐	☒	For null hypothesis testing, the test statistic (e.g. *F*, *t*, *r*) with confidence intervals, effect sizes, degrees of freedom and *P* value noted<br>*Give P values as exact values whenever suitable.*
☒	☐	For Bayesian analysis, information on the choice of priors and Markov chain Monte Carlo settings
☒	☐	For hierarchical and complex designs, identification of the appropriate level for tests and full reporting of outcomes
☒	☐	Estimates of effect sizes (e.g. Cohen's *d*, Pearson's *r*), indicating how they were calculated

Our web collection on statistics for biologists contains articles on many of the points above.

Software and code

Policy information about availability of computer code

Data collection	Cell counts for spleen samples were obtained on a Guava easyCyte (Luminex, Austin, TX), and cells were analyzed on an LSRFortessa flow cytometer (BD Biosciences). ELISpots were counted and data were analyzed using ImmunoSpot software (Cellular Technology Limited, Cleveland, OH).
Data analysis	All statistical analyses were conducted using Prism 9 (GraphPad Software, San Diego, CA).

For manuscripts utilizing custom algorithms or software that are central to the research but not yet described in published literature, software must be made available to editors and reviewers. We strongly encourage code deposition in a community repository (e.g. GitHub). See the Nature Portfolio guidelines for submitting code & software for further information.

Data

Policy information about availability of data

All manuscripts must include a data availability statement. This statement should provide the following information, where applicable:

- Accession codes, unique identifiers, or web links for publicly available datasets
- A description of any restrictions on data availability
- For clinical datasets or third party data, please ensure that the statement adheres to our policy

The datasets generated during and/or analyzed during the current study are available from the corresponding author on reasonable request.

Human research participants

Policy information about studies involving human research participants and Sex and Gender in Research.

Reporting on sex and gender	This study did not involve human research participants.
Population characteristics	This study did not involve human research participants.
Recruitment	This study did not involve human research participants.
Ethics oversight	This study did not involve human research participants.

Note that full information on the approval of the study protocol must also be provided in the manuscript.

Field-specific reporting

Please select the one below that is the best fit for your research. If you are not sure, read the appropriate sections before making your selection.

☒ Life sciences ☐ Behavioural & social sciences ☐ Ecological, evolutionary & environmental sciences

For a reference copy of the document with all sections, see nature.com/documents/nr-reporting-summary-flat.pdf

Life sciences study design

All studies must disclose on these points even when the disclosure is negative.

Sample size	Statistical analyses have been the guiding strategy to maximize statistical power while reducing the number of animals used to a minimum. We have previously determined that 6-8 animals per group (3-4 male, 3-4 female) is the lowest estimate to achieve meaningful comparisons between vaccine candidates with subtle differences in immunogenicity. 10 mice/group, 5 male and 5 female, is required to provide statistical power of 90% with an alpha (p-value) of 0.05 to detect a statistically significant difference in pseudovirus neutralizing antibody titers between vaccinated groups with two-fold differences in mean antibody titers with expected statistical variance. We use both male and female mice to allow for identification of any sex-specific differences in vaccine immunogenicity. For vaccine stability studies, n=5 all-female mice are used per group to provide statistical power of 80% to detect two-fold differences in serum antibody levels. The use of all female mice reduces data variability sufficiently to minimize animal group sizes across a very large study involving several hundred mice, while not affecting the main study readout of vaccine stability.
Data exclusions	No data were excluded from any plots or analyses.
Replication	Replication of data was conducted whenever possible. All in vitro assays were conducted with a minimum of biological triplicates. Bridging groups were used between independent animal studies to provide replication of key study groups across multiple studies offset by months. All attempts at replication verified the robustness of the scientific approach.
Randomization	Mice were randomized into study groups prior to study onset.
Blinding	Investigators were not blinded to the study groups during data collection and analysis as this is typically unnecessary for this type of inbred animal study and typically introduces more systemic and human error than it prevents. Quantitative, unbiased assays were developed with highly regulated SOPs in order to minimize data biases.

Reporting for specific materials, systems and methods

We require information from authors about some types of materials, experimental systems and methods used in many studies. Here, indicate whether each material, system or method listed is relevant to your study. If you are not sure if a list item applies to your research, read the appropriate section before selecting a response.

Materials & experimental systems

n/a	Involved in the study	
☐	☒	Antibodies
☐	☒	Eukaryotic cell lines
☒	☐	Palaeontology and archaeology
☐	☒	Animals and other organisms
☒	☐	Clinical data
☒	☐	Dual use research of concern

Methods

n/a	Involved in the study	
☒	☐	ChIP-seq
☐	☒	Flow cytometry
☒	☐	MRI-based neuroimaging

Antibodies

Antibodies used	The following antibodies were used: SARS-CoV-2 neutralizing monoclonal antibody (mAb; GenScript, Piscataway, NJ; #A02057); an Anti-Mouse IgG (Fc Specific)-Alkaline Phosphatase antibody (Sigma-Aldrich, #A2429); an IgG1 SARS-CoV-2 neutralizing antibody (GenScript #A02055); an IgG2a SARS-CoV-2 neutralizing antibody (GenScript #BS-M0220); CD28 costimulatory antibody (BD Biosciences #553294); CD16/CD32 antibody (Invitrogen #14-0161-86); IFNγ (BD Biosciences, #51-2525KZ), IL-17A (Invitrogen, #88-7371-88), and IL-5 (BD Biosciences, #51-1805KZ) ELISPOT capture antibodies; detection antibodies (IFNγ (BD Biosciences, #51-1818KA), IL-17A (Invitrogen, #88-7371-88), and IL-5 (BD Biosciences, #51-1806KZ)); secondary antibodies (Goat Anti-Mouse IgG-HRP (SouthernBiotech, #1030-05); IgA-HRP (SouthernBiotech #1040-05)); Cy3-conjugated rabbit polyclonal antibody to SARS-CoV-2 spike (Abcam #AB272504), costimulatory CD28 antibody (BD Biosciences #553294); flow cytometry surface stain antibodies (CD4 PerCP-Cy5.5 (eBioscience #45-0042-82), CD8 BV510 (BD Biosciences #563068CD44), CD44 APC-Cy7 (BD Biosciences #560568), and CD107a APC (Biolegend #121614); and flow cytometry intracellular staining antibodies (TNFa BV421 (Biolegend #506327), IL-2 PE-Cy5 (Biolegend #503824), IFNg PE-Cy7 (Invitrogen #25731182), IL-5 PE (eBioscience #12-7052-82), IL-10 BV711 (BD Biosciences #564081), and IL-17a AF700 (BD Biosciences #560820).
Validation	Each commercial antibody was validated by the manufacturer for the intended use, followed by an in-house AAHI validation for the intended purposes. Antibody concentrations appropriate for each assay were determined using experimental titration curves of each individual antibody in each assay.

Eukaryotic cell lines

Policy information about cell lines and Sex and Gender in Research

Cell line source(s)	HEK-293 cells (American Type Culture Collection [ATCC], Manassas, VA; #CRL-3216) were used in this study.
Authentication	These cell seed stocks were recently (within the last two years) obtained directly from ATCC, expanded and frozen at low passage (<p6), and used only at low passage number (<p20). Regular cell culture visualization was conducted to confirm cell morphology and size as expected. Additional authentication was not conducted.
Mycoplasma contamination	Cell lines were obtained mycoplasma-free directly from ATCC. Cultures were only used at low passage number to minimize risk of mycoplasma contamination. No further mycoplasma testing was routinely conducted.
Commonly misidentified lines (See ICLAC register)	*Name any commonly misidentified cell lines used in the study and provide a rationale for their use.*

Animals and other research organisms

Policy information about studies involving animals; ARRIVE guidelines recommended for reporting animal research, and Sex and Gender in Research

Laboratory animals	C57BL/6J mice obtained from The Jackson Laboratory (Harbor, ME) were used for all animal studies in this work.
Wild animals	The study did not involve wild animals.
Reporting on sex	Equal numbers of both male and female mice were used for all mouse studies in this work, with the sole exception of all female mice for the Figure 6 vaccine stability studies alone.
Field-collected samples	The study did not involve samples collected from the field.
Ethics oversight	All animal work was done under the oversight of the Bloodworks Northwest Research Institute's Institutional Animal Care and Use Committee (Seattle, WA).

Note that full information on the approval of the study protocol must also be provided in the manuscript.

Flow Cytometry

Plots

Confirm that:

☒ The axis labels state the marker and fluorochrome used (e.g. CD4-FITC).

☒ The axis scales are clearly visible. Include numbers along axes only for bottom left plot of group (a 'group' is an analysis of identical markers).

☒ All plots are contour plots with outliers or pseudocolor plots.

☒ A numerical value for number of cells or percentage (with statistics) is provided.

Methodology

Sample preparation	Spleens from C57BL/6J mice were dissociated in 4 mL of RPMI medium by manual maceration through a cell strainer using the end of a syringe plunger. Homogenized samples were briefly centrifuged at 400 x g (15-20 sec) to pellet fat cells. Samples

were then carefully resuspended and fat clumps removed by pipette. The supernatants containing lymphocytes were transferred to 5-mL mesh-cap tubes to strain out any remaining tissue debris or were lysed with ammonium-chloride-potassium (ACK) buffer and washed. Cell counts for each sample were obtained on a Guava easyCyte (Luminex, Austin, TX). Each spleen sample was seeded in 96-well round-bottom plates at 1-2 x 106 cells per well in RPMI medium containing 10% FBS, 50µM beta-mercaptoethanol, CD28 costimulatory antibody (0.4 µL/test, BD Biosciences #553294), and brefeldin A. Cells were stimulated with one of three stimulation treatments: 0.0475% dimethyl sulphoxide (DMSO) as a negative stimulation control, 0.2 µg/well (1 µg/mL) per peptide of spike peptide pool (JPT Peptide Technologies, Berlin, Germany; #PM-WCPV-S-1) in an equivalent amount of DMSO, or 10 µg/well of phorbol myristate acetate (PMA)/ionomycin solution. After 6 hours of incubation at 37°C with 5% CO2, plates were centrifuged at 400 x g for 3 minutes, the supernatants were removed by pipetting, and cells were resuspended in PBS. Plates were centrifuged, the supernatants were removed, and cells were stained for flow cytometry. Splenocytes were stained for viability with Zombie Green (BioLegend, San Diego, CA) in 50 µL of PBS, and then Fc receptors were blocked with CD16/CD32 antibody (0.25 µL/test, Invitrogen #14-0161-86). Cells were then surface stained with fluorochrome-labeled mAbs specific for mouse CD4 (0.3 µL/test, PerCP-Cy5.5, eBioscience #45-0042-82), CD8 (2 µL/test, BV510, BD Biosciences #563068CD44), CD44 (0.2 µL/test, APC-Cy7, BD Biosciences #560568), and CD107a (2 µL/test, APC, BioLegend #121614) in 50µL of staining buffer (PBS with 0.5% bovine serum albumin and 0.1% sodium azide). Cells were washed twice, permeabilized using the Fixation/Permeabilization Kit (BD Biosciences, Franklin Lakes, NJ), and stained with fluorochrome-labeled mAbs specific for mouse TNFα (2 µL/test, BV421, BioLegend #506327), IL-2 (0.4 µL/test, PE-Cy5, BioLegend #503824), IFNγ (0.2 µL/test, PE-Cy7, Invitrogen #25731182), IL-5 (0.7 µL/test, PE, eBioscience #12-7052-82), IL-10 (2 µL/test, BV711, BD Biosciences #564081), and IL-17A (2 µL/test, AF700, BD Biosciences #560820). After two washes in staining buffer, cells were resuspended in 100 µL of staining buffer and analyzed on an LSRFortessa flow cytometer (BD Biosciences).

Instrument

LSRFortessa flow cytometer (BD Biosciences)

Software

BD FACSDiva software was used for flow cytometric data analyses.

Cell population abundance

Samples were collected from bulk splenocytes, purified as described above, and stimulated for 6 hours prior to staining. For each sample, total events up to 1,000,000 were recorded, with samples generally having ~250,000-600,000 events, though this varies. Of these, approximately 70% are within the lymphocyte gate (~210,000), and of these 50% are within the live cell gate (110,000). The CD4 gate is ~25% of the parent population and the CD8 gate ~15% of the parent population. IFNg, IL-2 and TNFa represent 0-4% of the CD4 or CD8 cells. In the peptide-stimulated populations, CD107a is ~5-10% of the cells, IL-5 is 0-5% of cells, and IL-10 and IL-17 are generally 0-2% of the cells. Levels are close to 0 for most markers in the DMSO-stimulated wells and up to 50% in PMA-stimulated wells.

Gating strategy

All cells were gated first on the lymphocyte population, defined in the FSC/SSC plot to exclude cell debris, defined as FSC-A larger than 50K and SSC-A as greater than 25K generally, broadly tracing the outlines of the remaining cells. Next, live CD44 cells were gated with Zombie green on the Y-axis and CD44-Apc-Cyt on the X-axis. Live cells were defined as FITC (below 10^3 on the Y-axis) and CD44+ (~>200 on the X-axis). These cells were then gated on CD4-Percp-Cy5.5 (Y-axis) and CD8-BV510 (X-axis). CD4 cells were defined as CD4+CD8-, generally falling above 10^3 in CD4 and <2000 on CD8. CD8 cells were defined as CD4-CD8+, falling at below 10^3 on CD4 and above 2000 on the CD8 axis. Each of these populations was analyzed independently, gating for IFNg, IL-2, TNFa, CD107a, IL-5, and IL-10 in the histogram view, determining positive populations via the positive (PMA-stimulated) and negative (DMSO-stimulated) groups to set the individual histogram gates. Percent of polyfunctional cells was calculated using the Boolean-AND function in flowjo, including IFNg+, IL-2+, and TNFa+ CD4 or CD8 cells.

☒ Tick this box to confirm that a figure exemplifying the gating strategy is provided in the Supplementary Information.
